# Design and predict the potential of imidazole-based organic dyes in dye-sensitized solar cells using fingerprint machine learning and supported by a web application

**DOI:** 10.1038/s41598-024-76739-6

**Published:** 2024-11-03

**Authors:** Mohamed M. Elsenety

**Affiliations:** Department of Chemistry, Faculty of Science, Al-Azhar University, Nasr City, Cairo 11884 Egypt

**Keywords:** Imidazole, Organic dyes, Dye-sensitized solar cells, Machine learning, PCE prediction, Energy science and technology, Solar energy, Photovoltaics, Solar cells

## Abstract

This scientific paper presents a novel approach to explore and predict the potential of imidazole-based organic dyes for use in Dye-Sensitized Solar Cells (DSSCs) using a machine learning web application. The design of efficient and cost-effective organic dyes is critical to enhance the performance of DSSCs. Traditional experimental methods are time-consuming and resource-intensive, making it challenging to screen a large number of potential dyes. In this study, we propose a machine learning-based approach to accelerate the discovery process by predicting the photovoltaic performance of imidazole-based organic dyes. Machin learning predictions provide valuable insights into the expected PCE% and behaviors of the molecules toward DSSCs. Based on the RDKit library, several fingerprints such as Molecular ACCess System, Avalon, Daylight, Pharmacophore and Morgan with different radius (r2, r3, r4), were studied. In addition, more than 20 ML algorithms using different cross validation (3, 5, 7, 10) were also evaluated. Among of these, Deep Neural Network models of MLPRegressor algorithm based on the daylight fingerprint shows a significant coefficient of determination combined with the lowest errors. Utilize the trained ML models to screen of 50 million SMILE structure for identify promising imidazole and nitrogen-containing derivative as a doner group. By replacing the donor groups in the well-known MK2 dye structure with the top imidazole derivatives proposed by machine learning, significant improvements in PCE were observed, increasing from 7.70% to as high as 11.49%, representing nearly a 50% enhancement over the control. DFT calculations confirm the ML predictions and clarify the significantly higher oscillator strength and charge transfer properties of MK2-DM1, compared to MK2. This result provides a promising pathway for developing new dye materials that can push the efficiency limits of DSSCs, leading to more efficient solar energy conversion technologies in the future. In addition, a developed web application offers a user-friendly interface for researchers to input their molecular structures and obtain PCE% predictions toward DSSCs. This information can guide researchers in designing a new imidazole dye with high photovoltaic performance to validate and refine the predictions without time consuming.

**Supplementary Information:**

The online version contains supplementary material available at 10.1038/s41598-024-76739-6.

## Introduction

DSSCs have emerged as a promising alternative to traditional silicon-based solar cells due to their ease of fabrication, cost-effectiveness, and potential for flexible applications^1–3^. The efficiency of solar cells highly depends on the light-harvesting capabilities and electron injection efficiency of the sensitizing organic dyes^4–10^. Imidazole-based organic dyes have been extensively studied and used as sensitizers in DSSCs due to their promising optical and electronic properties^11–13^. Imidazole-based dyes often possess a conjugated structure, which allows them to absorb light across a wide range of wavelengths. This characteristic is essential for efficiently capturing sunlight and enhancing the efficiency of DSSCs. The absorption spectrum of imidazole-based dyes can be tuned by modifying their chemical structure. This tunability enables researchers to design dyes that match the solar spectrum and optimize light absorption. Imidazole-based dyes can facilitate efficient injection of electrons from the excited dye molecule into the semiconductor’s conduction band, which is a crucial step for generating a photocurrent^13^. Imidazole derivatives can be synthesized with various functional groups, allowing researchers to modify their properties and improve their performance in DSSCs. Imidazole-based dyes often exhibit good thermal and photochemical stability, which is important for maintaining the long-term performance of DSSCs under different operating conditions^14,15^. Organic dyes are typically less toxic and environmentally friendly compared to some inorganic materials used in solar cells. Organic dyes are generally less expensive to synthesize than some inorganic alternatives, which can contribute to making DSSCs more economically viable. Examples of imidazole-based organic dyes that have been investigated for use in DSSCs include those containing push–pull systems, anchoring groups, and other functional groups that enhance light absorption, electron injection, and stability. The identification and design of suitable dyes are crucial for enhancing the overall performance of DSSCs. And it well known, Experimental synthesis and characterization of new molecules can be time-consuming, expensive and reducing the resources. However, the Computational predictions can help researchers identify the most promising candidates, reducing the need for trial-and-error approaches and minimizing resource wastage^16,17^. Currently, the integration of Machine Learning (ML) has become a significant technique in the field of organic compounds for DSSCs^18^. ML leverages the power of data-driven analysis to expedite the discovery and design of novel organic compounds with enhanced performance characteristics. By training ML models on vast datasets of known organic compounds and their properties, researchers can predict the behavior of new compounds without the need for extensive laboratory experiments. ML models can identify structural features and relationships within organic compounds that contribute to improved light absorption, electron injection efficiency, and stability in DSSCs. This data-driven approach enables researchers to pinpoint the most promising candidates for further exploration, significantly reducing the time and resources traditionally required for compound discovery^19,20^. In this work, we present we introduces a machine learning-based approach to assess the potential of imidazole-based organic dyes for use in DSSCs through a user-friendly web application. This study utilizes machine learning models to predict the photovoltaic performance of imidazole-based organic dyes, offering insights into their expected efficiency (PCE%) in DSSCs using various molecular fingerprints and machine learning algorithms to make predictions. These trained models were then used to screen over 50 million SMILE structures, identifying thousands of molecules with potential PCE%. Additionally, we developed a user-friendly web application (freely available) where researchers can input their molecular structures to obtain PCE% predictions, facilitating the design of high-performance imidazole compounds for DSSCs without the need for time-consuming experiments.

## Data collection

To build a robust dataset for training and evaluating the machine learning model, data was collected from 128 scientific articles published between 2010 and 2022. These papers specifically focused on the application of imidazole-based dye derivatives in DSSCs. The collected data of the molecular structures of the dyes were divided into three parts manually of doner groups, linker, and anchoring groups. The doner structures were extracted and converted into Simplified Molecular Input Line Entry System (SMILES) strings, a format that allows machine learning algorithms to process chemical structures as text-based representations. In addition to the chemical structures, key photovoltaic performance metrics of the DSSCs were also collected, including short-circuit current density (J_sc_) in mA/cm^2^, Open-circuit voltage (V_oc_) in volts, Fill factor (FF), Power conversion efficiency (PCE%). These parameters are essential for evaluating the performance of DSSCs analysis, and PCE% was used as target variables in the machine learning model to predict the efficiency of new doner imidazole derivatives. Table S1 presents the collected imidazole structures and their corresponding reference.

## Methodology

Our methodology involves a seven-step process. 1st step, a diverse dataset of SMILE structure of imidazole-based organic dyes molecules and their corresponding values of the photovoltaic performance PCE% is collected from existing experiments in literature. 2nd step, fingerprint generation including Molecular ACCess System (MACCS), Avalon (Av), Daylight, Pharmacophore and Morgan fingerprints were generated by breaking down a molecule’s structure into specific substructures or fragments, and then encoding the presence or absence of these fragments in a binary format^21^. These fragments can represent different types of chemical features, such as functional groups, ring systems, and bond arrangements. However, these fingerprints are often used for similarity searching and are particularly useful for virtual screening and compound clustering. Each fingerprint algorithm has its own advantages and limitations depending on the specific use case. In general, MACCS are a set of structural keys that encode the presence or absence of specific structural fragments or patterns within a molecule. Besides that, the Topological Torsion Fingerprints capture the topological aspects of molecular structures, emphasizing the distribution of bond angles and the presence of torsional angles. They are useful for distinguishing between stereoisomers and conformational isomers. Moreover, the Avalon fingerprint algorithm generates a structural fingerprint by systematically encoding the arrangement of atom pairs and bond pairs within a molecule. These fingerprints are designed to capture 2D and 3D structural information and are useful for similarity searching and scaffold hopping. Furthermore, Daylight fingerprints are based on hashing and are designed to handle very large molecular databases efficiently. Furthermore, pharmacophore fingerprints encode pharmacophoric features such as hydrogen bond donors, hydrogen bond acceptors, aromatic rings, etc. They are useful for ligand-based virtual screening and studying structure–activity relationships. 3rd step, once the molecular structure is broken down and encoded, a binary vector is created where each element corresponds to the presence or absence of a specific fragment. This binary vector serves as the descriptor, which captures the structural features of the imidazole organic dyes information are extracted and used as input features for the machine learning model. 4th step, the prepared fingerprint molecular structure dataset is split into a training set and a validation/testing set. The training set is used to train over 20 machine learning models of regression algorithms including (RandomForestRegressor (RF), GradientBoostingRegressor (GB), ExtraTreesRegressor (ET), AdaBoostRegressor (AB), BaggingRegressor (BR), Support Vector Regression (SVR), KNeighborsRegressor (KNN), Neural Network Architecture of Multi-Layer Perceptron Regressor (MLP), Extreme Gradient Boosting Regressor (XGB), Light Gradient Boosting Machine Regressor (LGB), CatBoostRegressor (CB), DecisionTreeRegressor (DT), Lasso, Ridge(Rid), ElasticNet, GaussianProcessRegressor, KernelRidge, IsotonicRegression, Partial Least Squares Regression (PLS), PassiveAggressiveRegressor (PA), HuberRegressor (HR), TheilSenRegressor, RANSACRegressor, ARDRegression, BayesianRidge(Bay), TweedieRegressor, and PoissonRegressor (PR)), to learn the relationship between the different fingerprints of molecular structures and the PCE% for each^22–24^. 5th step, during training, the model learns how different fragments or substructures encoded in the different fingerprints correlate with the target molecular property. In addition, the dataset is divided into k subsets of roughly equal size using K-Fold Cross-Validation technique of (3, 5, 7, and 10) for assessing and validating the performance of a predictive model^25,26^. Statistical and visualization were used to identify patterns and relationships that allow the model to predict the PCE% for each molecule. 6th step, once the model is trained, it is evaluated using the validation/testing set to assess its predictive performance. Common metrics used for evaluation include mean squared error, and coefficient of determination (R^2^). 7th step, finally after the model is trained and validated, it is used to predict the PCE% of new molecules. To make predictions, the molecular structure of the new molecule is transformed into daylight fingerprints, which are then fed into the trained model to obtain a predicted value for the PCE%. However, we employed Python, utilizing open-source libraries including, pandas, matplotlib, pillow, seaborn, scikit-learn, scipy, and streamlit, to preprocess, visualize, conduct statistical analyses, and perform univariate, bivariate, and correlation analyses. The focus of our modeling efforts was on predicting the power conversion efficiency (PCE%) of the DSSCs devices incorporating the imidazole organic compounds. The machine learning web application is developed using python code and streamlit cloud to provide an intuitive and user-friendly platform for researchers in the field of DSSCs to access and utilize the predictive model. The web application allows users to input the molecular structure of their proposed imidazole-based organic dye and obtain predictions for key performance parameters. The output includes information about light absorption efficiency, electron injection efficiency, and charge transport properties, which are critical indicators of the dye’s potential as a sensitizer in DSSCs.

Density Functional Theory (DFT) calculations were performed using Gaussian 09 to evaluate the molecular orbitals, as well as the hole and electron reorganization energies of the dye molecules MK2 and MK2-DM1. The calculations were conducted using the PM6 semi-empirical method, which is suitable for providing a balance between computational efficiency and accuracy at limited resources. Time-Dependent DFT (TD-DFT) calculations were also performed to investigate the excited states of the dyes and their contributions to the absorption spectra.

## Results and discussion

**Fig. 1:**
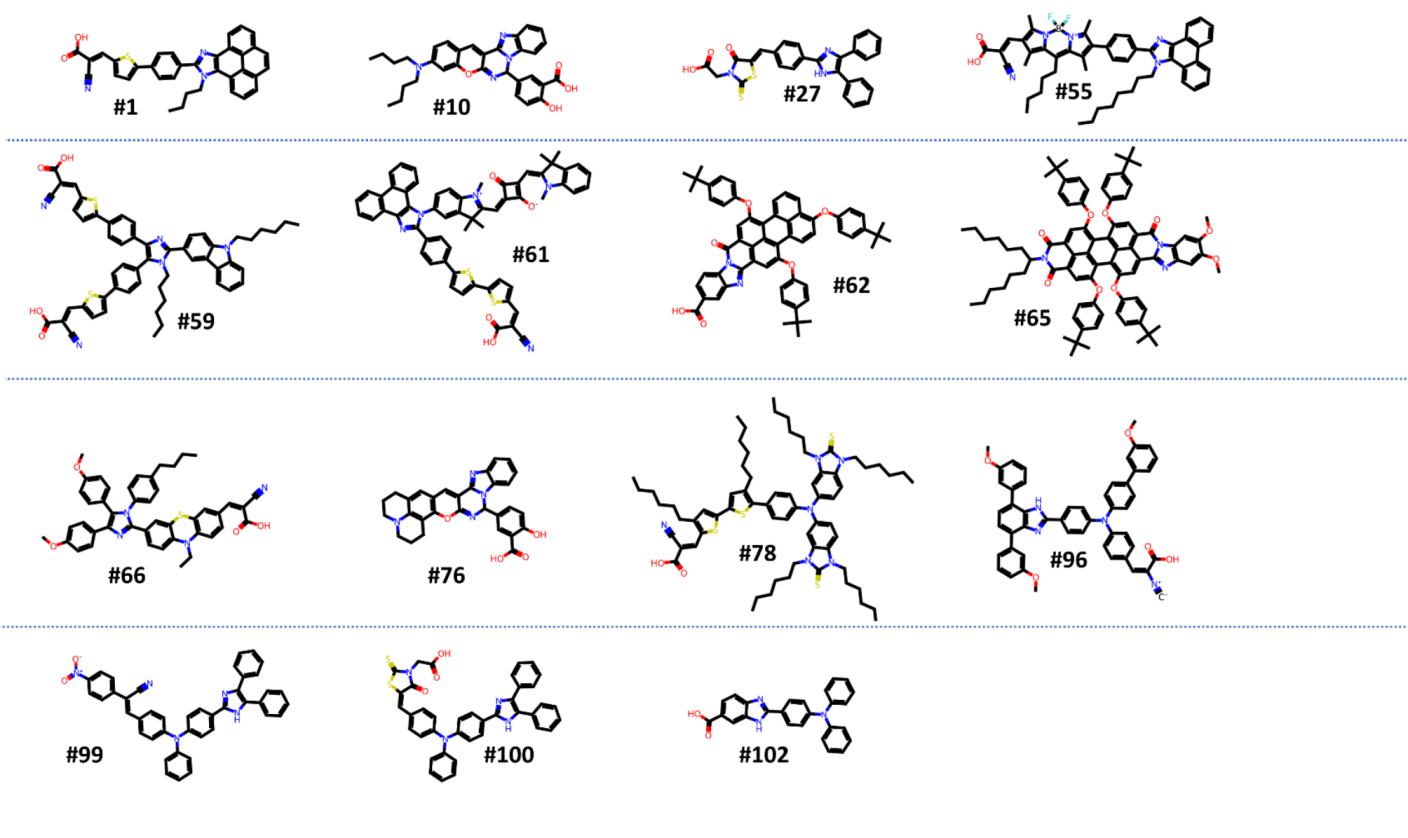
An example of 15 different types of the imidazole derivatives structures.

Imidazole-based derivatives have been explored as organic dyes in DSSCs due to their interesting electronic properties and potential for efficient light absorption and electron injection. However, imidazole’s structure is a five-membered aromatic heterocycle with two nitrogen atoms in the ring. Its structure can be modified by adding various substituents to tailor its properties for use as a photosensitizer in DSSCs. Figure 1 shows different types of imidazole derivatives structures of organic dyes which collected from existing experiments in literature. The imidazole derivatives were classified into 15 categories based on their anchoring and donor groups (Tables S1 and S2), allowing for easy identification of variations in these groups across the compounds. It’s worth mentioning, all compounds have imidazole moiety as a common donor group. However, some additional donor groups were also found beside imidazole moiety such as Bodipy, Perylene/Anthracene, Carbazole, Indoline/Squaraine, Nitro, Phenothiazine and Triphenylamine. While the anchoring groups of compounds include carboxylic acid, cyanoacrylic, carboxylic/rhodamine, carboxylic/rhodamine, nitro and benzoic/carboxylic groups.

**Fig. 2:**
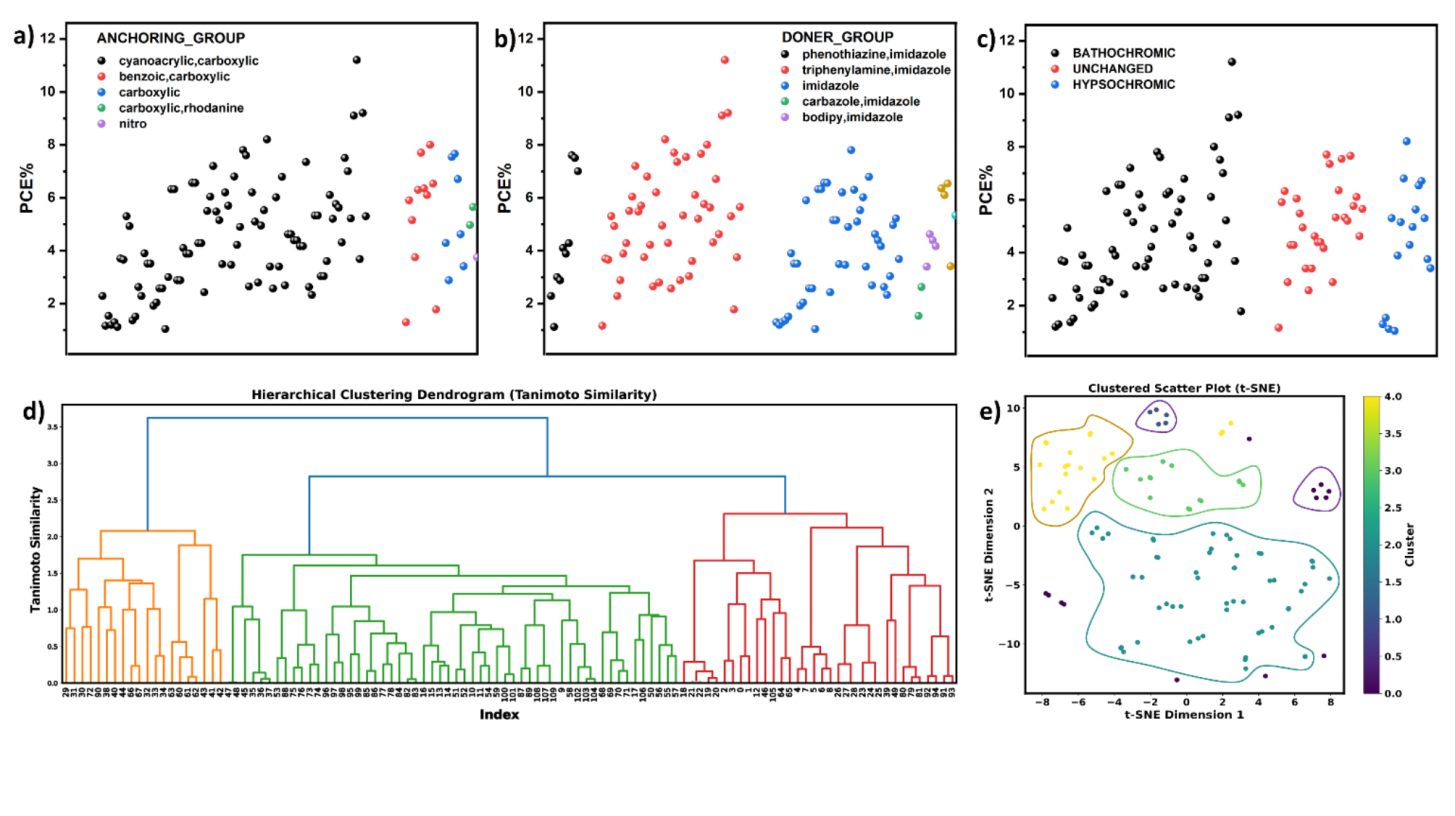
Photovoltaic performance toward anchoring (**a**), or doner (**b**)groups, and bathochromic shift (**c**) of imidazole derivatives. Tanimoto similarity of hierarchical clustering dendrogram visualization (**d**) or using t-distributed Stochastic Neighbor Embedding technique (**e**).

Figure 2a,b show a significant positive correlation between PCE (%) and Cyanoacrylic/carboxylic or Triphenylamine/imidazole derivatives as anchoring or doner groups, respectively. As expected, achieving a bathochromic shift (Fig. 2c) refers to the ability of imidazole derivatives to absorb light at lower energy of longer wavelengths. This shift allows the imidazole organic compounds to capture a larger portion of the solar spectrum, potentially increasing the overall efficiency of the DSSCs.

However, when applying hierarchical clustering, using the Tanimoto similarity metric, the molecules were grouped based on their structural similarities resulted in the consolidation of the original fifteen categories into three primary clusters, visualized in the dendrogram Fig. 2d. In general, the y-axis represents the “distance” between clusters of molecules^26,27^. This distance metric is a measure of dissimilarity between clusters. The dendrogram is constructed by iteratively merging clusters based on their similarity. Thus, lower values of Tanimoto similarity indicate that clusters were merged at a smaller distance, implying higher similarity or proximity between those clusters. As you move up the y-axis, clusters with progressively higher distances were merged, suggesting less similarity.

**Fig. 3:**
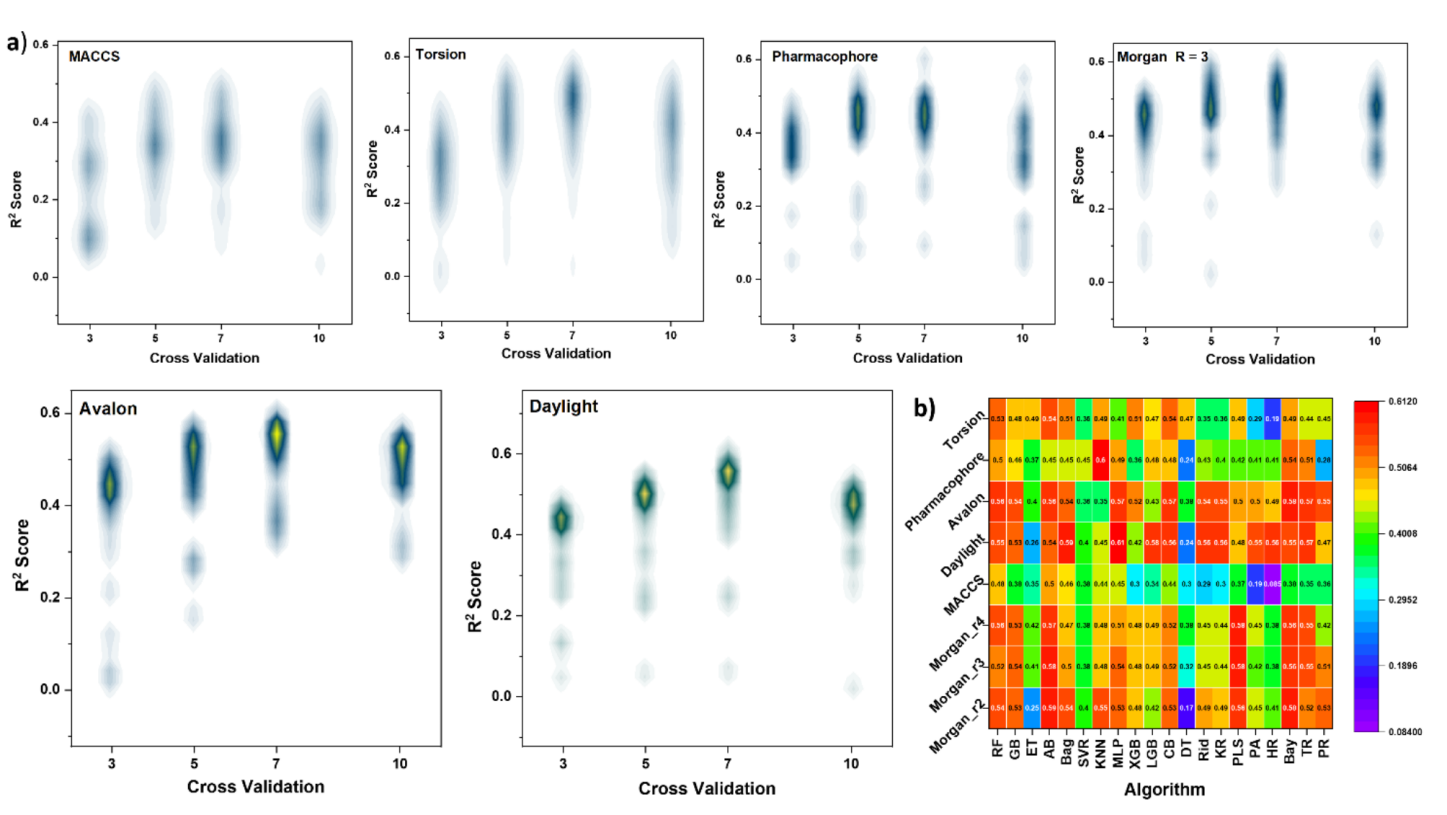
cross-validation for each fingerprint using different values of k-fold cross-validation (3, 5, 7, and 10).

For the simplest, dimensionality reduction techniques of t-distributed Stochastic Neighbor Embedding (t-SNE) were used to reduce the high-dimensional fingerprint data. Thus, the scatter plot of the colored points (Fig. 2e) based on the clusters identified by hierarchical clustering shows the similarity of compounds could classified to three main categories instead of fifteen categories. Table S3 provides a quantitative measure of the similarity or dissimilarity between pairs of clusters in a hierarchical clustering analysis of molecular data. It offers valuable information for characterizing the structural and chemical relationships between clusters of molecules. Moreover, the Tanimoto similarity distance shows how it relates to the relationships between the clusters. Clusters with low distances (e.g., Cluster 1 vs. Cluster 109) are more closely related, indicating similar chemical properties. While clusters with moderate distances (e.g., Cluster 4 and Cluster 48) represent intermediate levels of similarity. In contrast, clusters (e.g., Cluster 213 vs. Cluster 215) with high distances are less related and may correspond to structurally distinct groups. Subsequently, we employ various fingerprint generation techniques, including MACCS, Avalon, Daylight, Pharmacophore, and Morgan fingerprints. These fingerprints deconstruct the molecular structures into specific substructures or fragments and encode their presence or absence in a binary format. Different fingerprints capture distinct chemical features such as functional groups, ring systems, and bond arrangements. Each fingerprint algorithm offers its unique advantages and limitations tailored to specific use cases. However, more than 20 regression models using various algorithms were evaluated. The code performs cross-validation for each model using different values of k-fold (3, 5, 7, and 10). The best-performing models for each fingerprint based on the R^2^ score show significant values at k-fold = 7 of cross-validation as presented in Fig. 3a.

It’s well known, choosing the right algorithm and fingerprint representation is crucial for molecular property prediction. Interestingly, the analysis of molecular property prediction of different machine learning algorithms’ performance based on various molecular fingerprint representations was presented using heat mapping in Fig. 3b**.** We noted that, the substantial impact of the chosen fingerprint representation on model performance. Different fingerprints exhibited varying degrees of compatibility with the machine learning algorithms, with fingerprints such as Daylight demonstrating broad applicability across multiple models, while Avalon appeared to be more specialized. This underscores the importance of careful consideration when selecting the most suitable fingerprint representation based on the specific molecular properties and characteristics of the dataset.

Table S4 of daylight (as example) Fingerprint models score, show for each model the corresponding k-fold and regression evaluation metrics concerning coefficient of determination (R^2^), mean absolute error (MAE), mean squared error (MSE), and root mean squared error (RMSE), which can be calculated by Scikit-class metrics according to Eqs. (1–4)^28,29.^

1 $$R^{2} = 1 - \frac{{\sum_{i} \left( {y_{i} - \hat{y}_{i} } \right)^{2} }}{{\sum_{i} \left( {y_{i} - \overline{y}} \right)^{2} }}$$

2 $$MAE = \frac{1}{N}\sum_{i = 1}^{N} \left| {y_{i} - \hat{y}_{i} } \right|$$

3 $$MSE = \frac{1}{N}\sum_{i = 1}^{N} \left( {y_{i} - \hat{y}_{i} } \right)^{2}$$

4 $$RMSE = \sqrt {\frac{{\sum_{i = 1}^{N} \left( {y_{i} - \hat{y}_{i} } \right)^{2} }}{N}}$$

where N is the number of recorded samples, $y_{i}$ is the predicted PCE% value, and $\hat{y}_{i}$ is the experimental PCE% value.

Among all models, the MLP and AB algorithm consistently demonstrated robust and reliable performance across all evaluated molecular fingerprint representations. However, MLP algorithm levels the highest R^2^ of 0.61 with daylight fingerprint. This finding suggests that MLP Regressor can serve as a dependable choice for predictive modeling in molecular property estimation, offering versatility and resilience in various chemical contexts. Moreover, our analysis highlighted the superiority of artificial neural network (ANN) methods, exemplified by MLP Regressor, over ensemble methods and single models like DT Regressor^30^. The results reveal that the MLP is better suited for more complex datasets, especially when there are non-linear relationships between features and target variables. Furthermore, we identified the potential for model enhancement through hyperparameter tuning. Fine-tuning the model parameters was further optimized to predictive performance, tailoring the model to specific molecular datasets and property prediction tasks. The MLP models learn the intricate relationships between the daylight molecular fingerprints and the corresponding PCE% values. MLP models gain insights into how various molecular fragments correlate with the target property (PCE%) using K-Fold Cross-Validation techniques (with k values of 7) to rigorously assess and validate model performance and predict the minimum errors and the coefficients of determination.

**Fig. 4:**
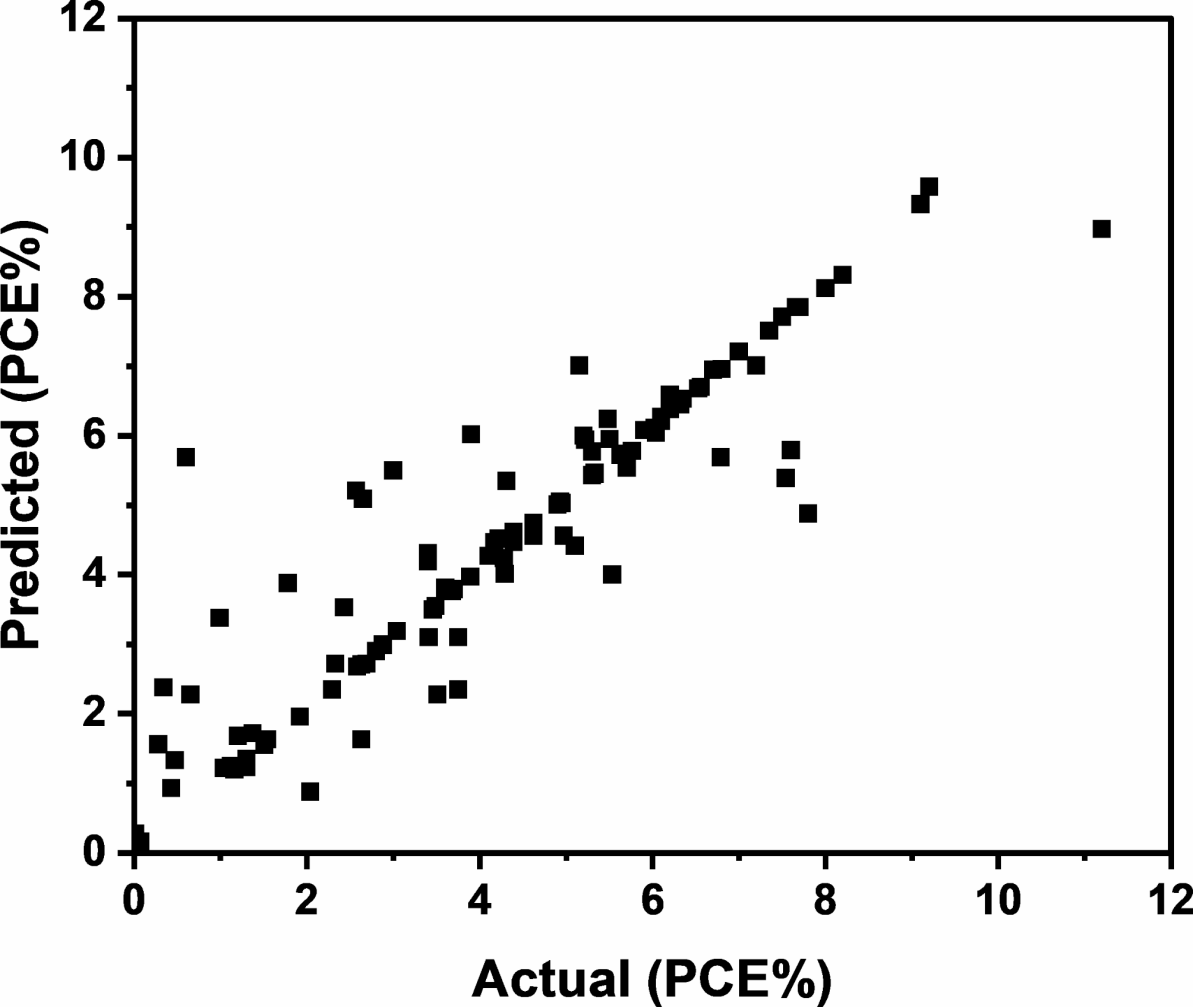
MLP regression model results of experimental and predicted PCE%.

Figure 4 indicates that the MLP regression model results of experimental and predicted PCE values with higher coefficient of determination and corresponding lower error values. This confirms that the MLP regression model fits better the experimental data and could be able to predict with higher accuracy the expected optimal PCE values. To make predictions, we transform the molecular structure of the new compound into daylight fingerprints. These fingerprints are then fed into the trained model to obtain precise predictions for the PCE%.

Over than 50 million smile structures containing nitrogen atoms from GDB-17 database^31^ were tested based on selected MLP Regressor algorithm and the daylight fingerprint to predict power conversion efficiency. The results show that 5492, 238, 6 molecules provide significantly higher PCE% over 6%, 7% and 8%, respectively. Interestingly, the results reveal that selected molecules could be a significant choice as doner group which can connected with an anchoring group through linker or conjugated system in design of dye organic molecules with higher PCE% than expected.

**Fig. 5:**
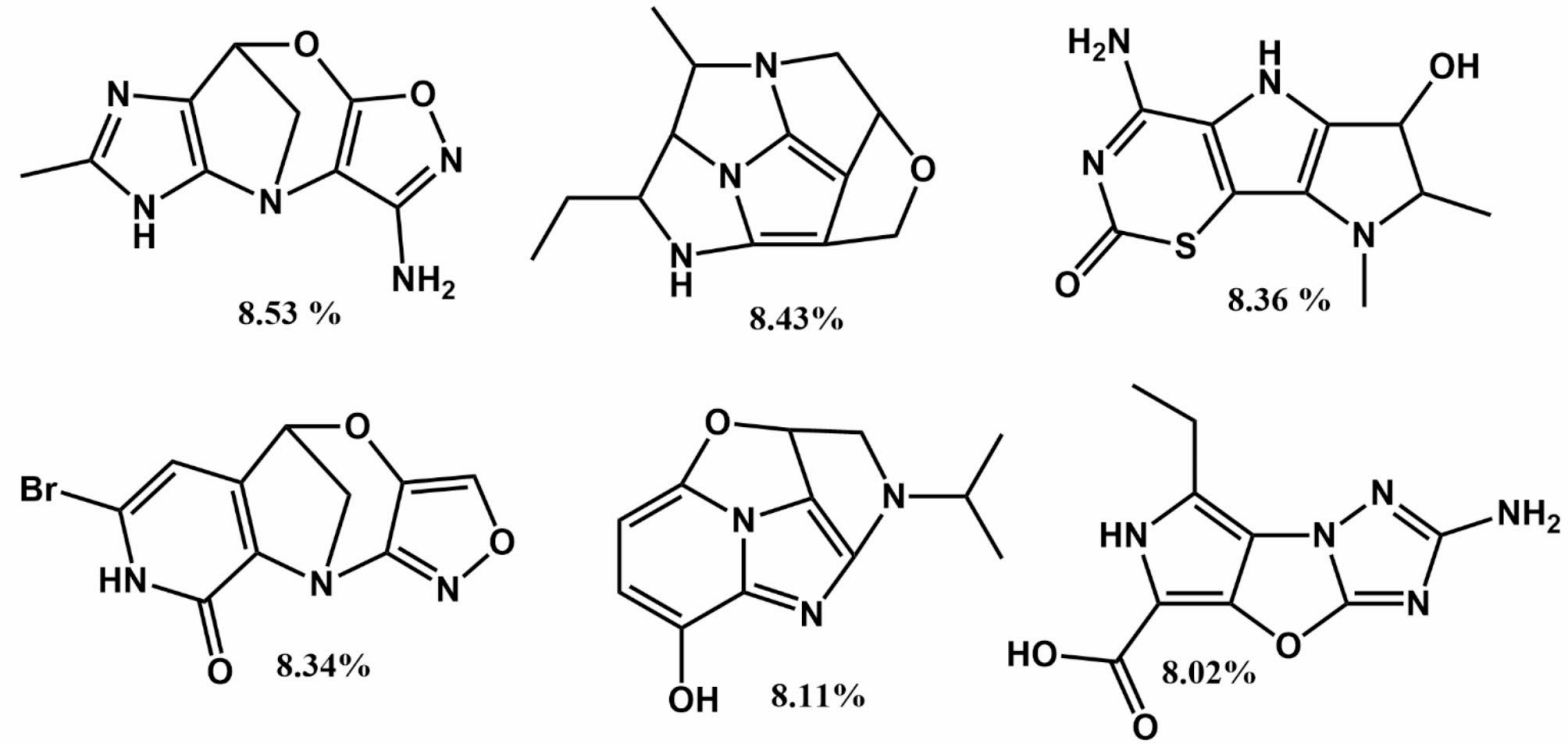
Top 6 imidazole derivatives with their predicted PCE%.

Further, the top 6 chemical structures with their predicted PCE% were shown in Fig. 5. However, 238 SMILE chemical structures with high predicted PCE% were selected and recorded in Table S5. Future research should consider the potential effects of that suggested chemical structure in the DSSCs application.

**Fig. 6:**
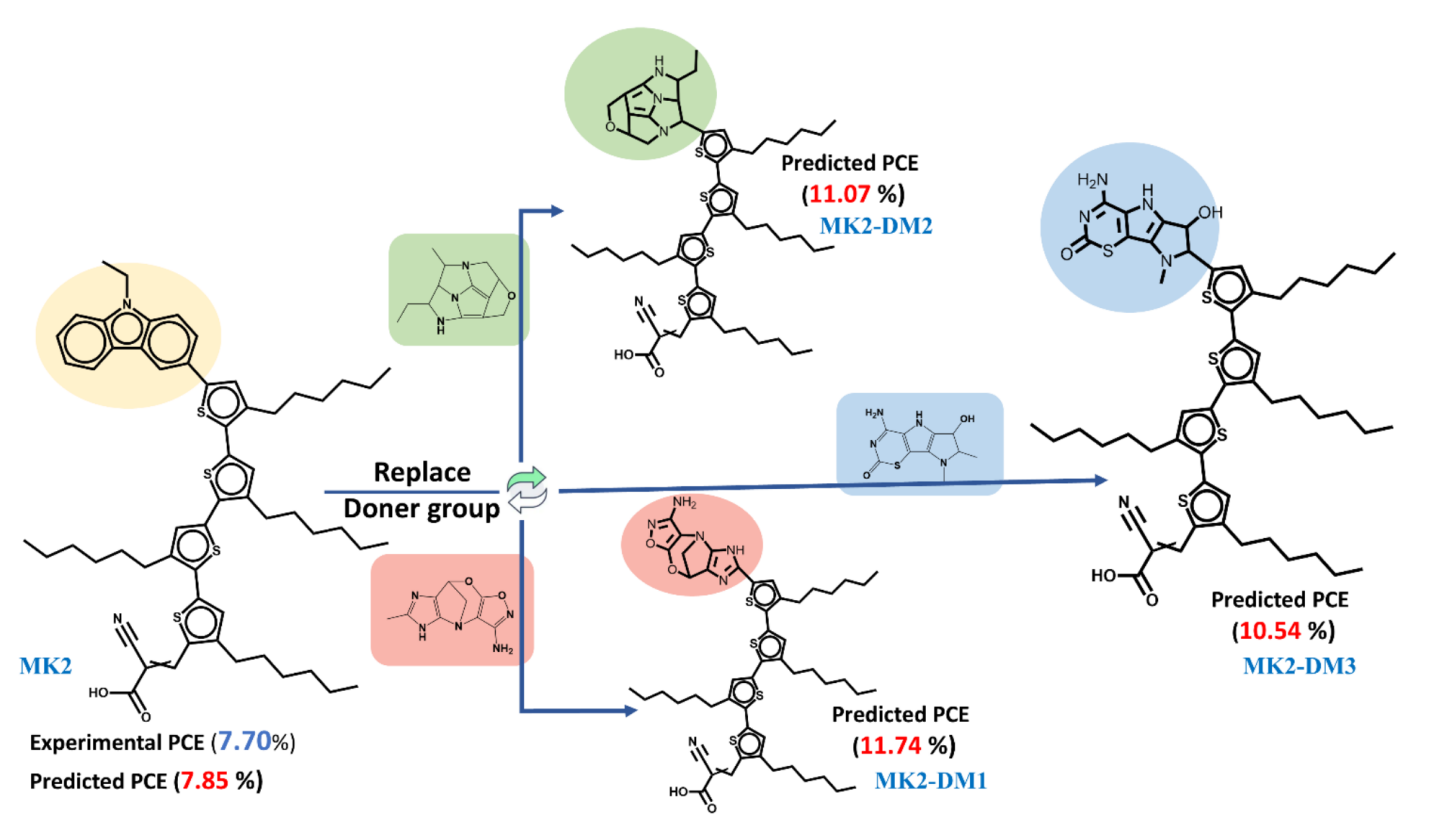
Predicted PCE% of replaced three top suggested chemical structure of imidazole derivative as a doner group (MK2-DM1, MK2-DM2, MK2-DM3) of one well known imidazole dye (MK2) from literature.

In addition, the study focused on replacing the existing donor group of the well-known MK2 dye^32^ with the top three predicted imidazole donor groups, as illustrated in Fig. 6. The results demonstrated a significant increase in the efficiency of the dye, showcasing the importance of donor group selection in enhancing photovoltaic performance. The experimental PCE of the MK2 dye was initially 7.07%, while the predicted PCE (based on modeling) was 7.85%. After replacing the donor groups with the top three proposed imidazole derivatives, the PCE improved to 11.74%, 11.07%, and 10.54%, respectively. This improvement represents a substantial enhancement of up to 49% compared to the initial efficiency of the MK2 dye. The substantial PCE increases observed when replacing the existing donor groups of the MK2 dye with these imidazole derivatives highlight their potential to serve as key components in next-generation DSSCs.

**Fig. 7:**
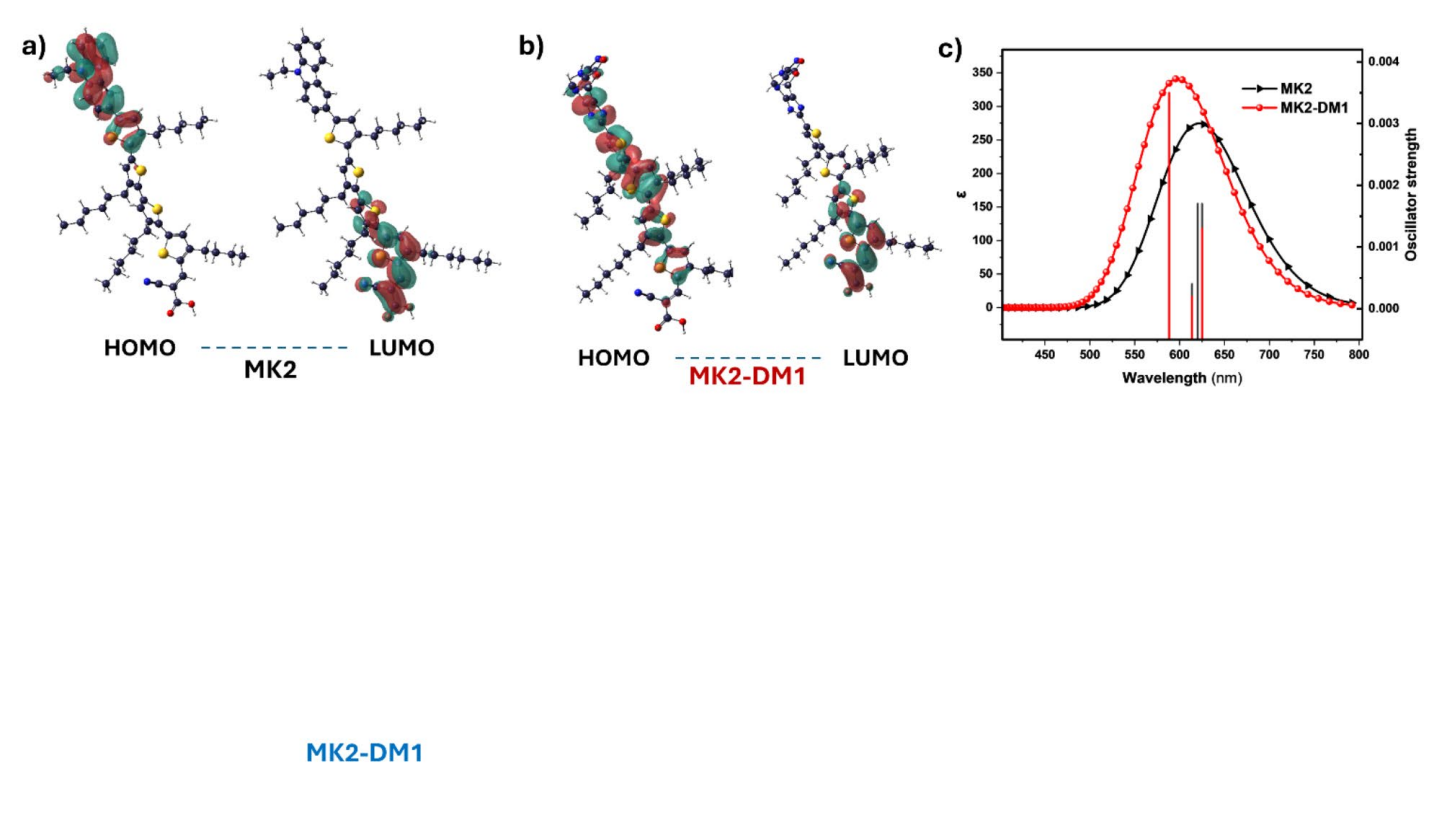
(**a**,**b**) HOMO and LUMO molecular orbital representation of both MK-2 (control) and MK2-DM1 (with a modified donor group), respectively; (**c**) TD-DFT calculated UV–Vis spectra for both compounds, MK-2 and MK2-DM1.

The computational analysis involves density functional theory (DFT) and time-dependent DFT (TD-DFT) calculations on MK-2 (the original, unmodified dye) and MK2-DM1 (MK-2 with a modified donor group). To ensure a fair comparison while saving computational resources, both compounds were optimized and analyzed using the same level of theory. This comparison aims to evaluate the impact of the donor group modification on the electronic and optical properties of the dye, which are crucial for dye-sensitized solar cell (DSSC) performance. Figure 7a,b shows HOMO and LUMO molecular orbital representation of both MK-2 and MK2-DM1, respectively. HOMO molecular orbital of both compounds focused on the doner groups with slightly better distribution on the modified structure of MK2-DM1 than control MK2, which favorable for intramolecular charge transfer from doner to acceptor group in the system. In addition, UV–vis. spectra of both compounds were calculated using TD-DFT as shown in Fig. 7c, and corresponding orbital contribution and in Table S6. It’s clear that both compounds have absorption peaks within the visible spectrum, but MK2 generally has a slightly red-shifted absorption compared to MK2-DM1. As well known, longer wavelength absorption is beneficial for harvesting more sunlight, particularly in the red and near-infrared regions, which can increase efficiency. However, MK2-DM1 has a significantly higher oscillator strength of 0.0035 at the peak at 588 nm, indicating stronger absorption at that wavelength compared to most peaks in MK2 of oscillator strength around 0.0017. This finding suggests that it have better overall absorption efficiency, which is beneficial for increasing the photocurrent in DSSCs, which also in excellent agreement with ML proposed.

In addition, electron and hole reorganization energy were investigated to determine the efficiency of charge transport processes. A lower reorganization energy indicates reduced structural reconfiguration during charge transfer, resulting in faster and more efficient electron and hole mobility^33,34^. In this context, MK2-DM1 exhibits lower reorganization energies (electron: − 0.2735 eV; hole: − 0.5627 eV), compere (electron: 4.133 eV; hole: 4.246 eV) of MK2, suggesting improved charge transfer properties. The significantly reduced reorganization energies values imply minimal structural relaxation, which leads to less resistance during charge mobility, thereby enhancing the overall DSSC efficiency.

**Fig. 8:**
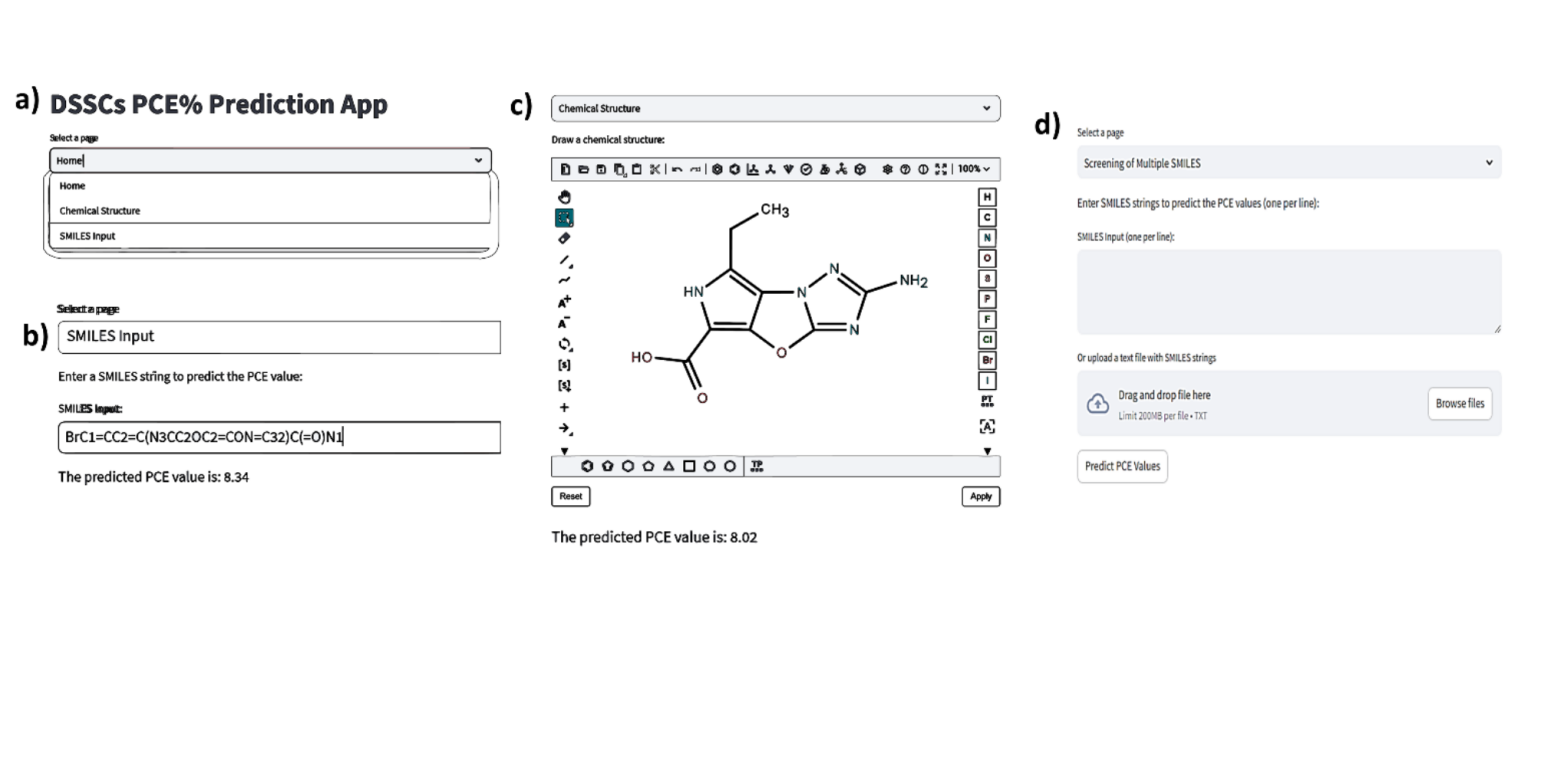
web application for DSSCs, (**a**) the main web page, (**b**) SMILE input page, (**c**) flexible and facile chemical structures drawing, (**D**) screening and evaluating multiple SMILES.

The machine learning web application^35^ was developed using python and streamlit cloud to provide user-friendly platform for researchers in the field of DSSCs to access and utilize the predictive model. The web application allows users to input the molecular structure of their proposed imidazole-based organic dye and obtain predictions for key performance parameters as shown in Fig. 8. However, we introduce three options for users to input their molecular structure, 1st is the SMILE format, and the 2nd is the flexible multi-optional page with all tools required to drawing the chemical structure, Lastly, the screening process involves evaluating multiple SMILES strings, either by importing them from a text file or entering them line by line into an input text box. This method provides an efficient and time-saving option for screening a large database of chemical structures, allowing for quick identification of potential candidates without the need for time-consuming manual input.

The machine learning web application is a groundbreaking tool designed with the utmost precision to cater to the specific needs of researchers delving into the realm of DSSCs. Its primary aim is to furnish these scientists with a seamlessly intuitive and user-friendly platform that empowers them to harness the potential of predictive models effectively. This innovative application operates on the principle of molecular structure analysis. Researchers can effortlessly input the intricate details of their imidazole-based organic dye compounds. In return, they receive comprehensive predictions that encompass vital performance metrics. By facilitating access to such critical data, this web application not only streamlines research but also accelerates the progress towards sustainable and efficient solar energy solutions.

## Conclusion

In this paper, we present a machine learning-based approach and a user-friendly web application for designing and predicting the potential of imidazole and nitrogen-contenting based organic dyes for DSSCs. The integration of machine learning and DFT calculations with DSSC application offers an efficient and cost-effective method to explore a vast chemical space and identify promising dye candidates. This study not only expedites the development of high-performance DSSCs but also opens up new possibilities for the design of organic materials tailored to specific solar cell requirements and could be a novel idea for other applications and bridging the gap between computational predictions and experimental validation.

## Future directions

The machine learning web application lays the foundation for further research in the field of solar cell materials discovery. Future directions should include expanding the dataset with more diverse and novel dye structures, incorporating more advanced machine learning techniques, and improving the model accuracy. However, researchers should consider the potential effects of that suggested chemical structure recorded in Table S3 as doner group of imidazole dye for the DSSCs application. Additionally, careful consideration should be given to the synthesizability of this chemical structure, including the complexity of the synthesis route, availability of starting materials, and the overall yield.

## Electronic supplementary material

Below is the link to the electronic supplementary material.

Supplementary Material 1

## Data Availability

The web application is freely available “https://elsenety.github.io/ml_imidazole_dssc.html” with the first version of model (V0.1), and the updated model will be available in same page. More information or any consideration is available on request due to privacy/ethical restrictions from the corresponding author.

## References

[1] Munukutla, L. V., Htun, A., Radhakrishanan, S., Main, L. & Kannan, A. M. *Dye-Sensitized Solar Cells* (Wiley, 2013). 10.1002/9781118845721.ch6

[2] Nalzala Thomas, M. R., Kanniyambatti Lourdusamy, V. J., Dhandayuthapani, A. A. & Jayakumar, V. Non-metallic organic dyes as photosensitizers for dye-sensitized solar cells: A review. *Environ. Sci. Pollut. Res. ***28**(23), 28911–28925. 10.1007/s11356-021-13751-7 (2021). 10.1007/s11356-021-13751-7 33856633

[3] Muñoz-García, A. B. et al. Dye-sensitized solar cells strike back. *Chem. Soc. Rev. ***50**(22), 12450–12550. 10.1039/d0cs01336f (2021). 34590638 10.1039/d0cs01336f PMC8591630

[4] Mao, M. et al. Highly efficient light-harvesting boradiazaindacene sensitizers for dye-sensitized solar cells featuring phenothiazine donor antenna. *J. Power Sources ***268**, 965–976. 10.1016/J.JPOWSOUR.2014.05.079 (2014).

[5] Luo, J. et al. N-annulated perylene as an efficient electron donor for porphyrin-based dyes: Enhanced light-harvesting ability and high-efficiency Co(II/III)-based dye-sensitized solar cells. *J. Am. Chem. Soc. ***136**(1), 265–272. 10.1021/JA409291G/SUPPL_FILE/JA409291G_SI_001.PDF (2014). 24345083 10.1021/ja409291g

[6] Rezk, H., Elsenety, M. M., Ferahtia, S., Falaras, P. & Zaky, A. A. A novel parameter identification strategy based on COOT optimizer applied to a three-diode model of triple cation perovskite solar cells. *Neural Comput. Appl. ***2023**, 1–23. 10.1007/S00521-023-08230-8 (2023).

[7] Elsenety, M. M. et al. Stability improvement and performance reproducibility enhancement of perovskite solar cells following (FA/MA/Cs)PbI_3-x_Br_x_/(CH_3_)_3_SPbI_3_ dimensionality engineering. *ACS Appl. Energy Mater. ***3**(3), 2465–2477. 10.1021/acsaem.9b02117 (2020).

[8] Kaltzoglou, A. et al. Synthesis, characterization and optoelectronic properties of chemically stable (CH_3_)_3_SPbI_3−x_Br_x_ and (CH_3_)_3_SPbI_3−x_Cl_x_ (X = 0, 1, 2, 3) perovskites. *Polyhedron ***140**, 67–73. 10.1016/j.poly.2017.11.030 (2018).

[9] Elsenety, M. M. et al. Synthesis, crystal structure, and broadband emission of (CH3)3SSnCl3. *Inorg. Chem. ***61**(11), 4769–4777. 10.1021/ACS.INORGCHEM.2C00181 (2022). 35254810 10.1021/acs.inorgchem.2c00181

[10] Christopoulos, E. et al. 3D/1D architecture using a 1-hexyl-3-methylimidazolium lead triiodide interlayer for robust and highly performing perovskite solar cells. *ACS Appl. Electron. Mater. *10.1021/ACSAELM.2C01783 (2023).

[11] Yadav, I. S. & Misra, R. Design, synthesis and functionalization of BODIPY dyes: Applications in dye-sensitized solar cells (DSSCs) and photodynamic therapy (PDT). *J. Mater. Chem. C Mater. ***11**(26), 8688–8723. 10.1039/D3TC00171G (2023).

[12] Zimosz, S. et al. New D-π-D-π-A systems based on phenothiazine derivatives with imidazole structures for photovoltaics. *J. Phys. Chem. C ***126**(21), 8986–8999. 10.1021/ACS.JPCC.2C01697/ASSET/IMAGES/LARGE/JP2C01697_0011.JPEG (2022).

[13] Nhari, L. M., El-Shishtawy, R. M., Bouzzine, S. M., Hamidi, M. & Asiri, A. M. Phenothiazine-based dyes containing imidazole with π-linkers of benzene, furan and thiophene: synthesis, photophysical, electrochemical and computational investigation. *J. Mol. Struct. ***1251**, 131959. 10.1016/J.MOLSTRUC.2021.131959 (2022).

[14] Pashaei, B., Shahroosvand, H., Graetzel, M. & Nazeeruddin, M. K. Influence of ancillary ligands in dye-sensitized solar cells. *Chem. Rev. ***116**(16), 9485–9564. 10.1021/ACS.CHEMREV.5B00621/ASSET/IMAGES/MEDIUM/CR-2015-00621Q_0093.GIF (2016). 27479482 10.1021/acs.chemrev.5b00621

[15] Park, S., Kwon, J. E. & Park, S. Y. Strategic emission color tuning of highly fluorescent imidazole-based excited-state intramolecular proton transfer molecules. *Phys. Chem. Chem. Phys. ***14**(25), 8878–8884. 10.1039/C2CP23894B (2012). 22618241 10.1039/c2cp23894b

[16] Kabanakis, A. N., Bidikoudi, M., Elsenety, M. M., Vougioukalakis, G. C. & Falaras, P. Synthesis of novel semi-squaraine derivatives and application in efficient dye-sensitized solar cells. *Dyes Pigm. ***165**, 308–318. 10.1016/j.dyepig.2019.02.028 (2019).

[17] Elsenety, M. M. et al. Synthesis, characterization and use of highly stable trimethyl sulfonium Tin(IV) halide defect perovskites in dye sensitized solar cells. *Polyhedron ***150**, 83–91. 10.1016/j.poly.2018.05.001 (2018).

[18] Elsenety, M. M., Christopoulos, E. & Falaras, P. Passivation engineering using ultrahydrophobic donor–π–acceptor organic dye with machine learning insights for efficient and stable perovskite solar cells. *Sol. RRL ***7**(10), 2201016. 10.1002/solr.202201016 (2023).

[19] Sutar, S. S. et al. Analysis and prediction of hydrothermally synthesized ZnO-based dye-sensitized solar cell properties using statistical and machine-learning techniques. *ACS Omega ***6**(44), 29982–29992. 10.1021/acsomega.1c04521 (2021). 34778669 10.1021/acsomega.1c04521 PMC8582059

[20] Li, F. et al. Machine learning (ML)-assisted design and fabrication for solar cells. *Energy Environ. Mater. ***2**(4), 280–291. 10.1002/EEM2.12049 (2019).

[21] Shaikh, F., Tai, H. K., Desai, N. & Siu, S. W. I. LigTMap: Ligand and structure-based target identification and activity prediction for small molecular compounds. *J. Cheminform. ***13**(1), 1–12. 10.1186/S13321-021-00523-1/TABLES/8 (2021). 34112240 10.1186/s13321-021-00523-1 PMC8194164

[22] Huang, J. C., Ko, K. M., Shu, M. H. & Hsu, B. M. Application and comparison of several machine learning algorithms and their integration models in regression problems. *Neural Comput. Appl. ***32**(10), 5461–5469 (2020).

[23] Doan, T. & Kalita, J. Selecting machine learning algorithms using regression models. In *Proceedings—15th IEEE International Conference on Data Mining Workshop, ICDMW 2015*, 1498–1505. 10.1109/ICDMW.2015.43

[24] Mitchell, J. B. O. Machine learning methods in chemoinformatics. *Wiley Interdiscip. Rev. Comput. Mol. Sci. ***4**(5), 468–481. 10.1002/WCMS.1183 (2014). 10.1002/wcms.1183 PMC4180928 25285160

[25] Mamat, N., Hamzah, F. M. & Jaafar, O. Hybrid support vector regression model and k-fold cross validation for water quality index prediction in Langat River, Malaysia. *bioRxiv*. 10.1101/2021.02.15.431242 (2021).

[26] Kibbey, C. & Calvet, A. Molecular property EXplorer: A novel approach to visualizing SAR using tree-maps and heatmaps. *J. Chem. Inf. Model. ***45**(2), 523–532. 10.1021/CI0496954 (2005). 15807518 10.1021/ci0496954

[27] Maccuish, J. D. & Maccuish, N. E. Chemoinformatics applications of cluster analysis. *Wiley Interdiscip. Rev. Comput. Mol. Sci. ***4**(1), 34–48. 10.1002/WCMS.1152 (2014).

[28] Elsenety, M. M. et al. Boosting perovskite nanomorphology and charge transport properties via a functional D–π-A organic layer at the absorber/hole transporter interface. *Nanoscale ***12**(28), 15137–15149. 10.1039/D0NR02562C (2020). 32638773 10.1039/d0nr02562c

[29] Elsenety, M. M., Mohamed, M. B. I., Sultan, M. E. & Elsayed, B. A. Facile and highly precise ph-value estimation using common PH paper based on machine learning techniques and supported mobile devices. *Sci. Rep. ***12**(1), 1–10. 10.1038/s41598-022-27054-5 (2022). 36585481 10.1038/s41598-022-27054-5 PMC9803664

[30] Ahmed, F. E. Artificial neural networks for diagnosis and survival prediction in colon cancer. *Mol. Cancer ***4**(1), 1–12 (2005). 16083507 10.1186/1476-4598-4-29 PMC1208946

[31] Ruddigkeit, L., Van Deursen, R., Blum, L. C. & Reymond, J. L. Enumeration of 166 billion organic small molecules in the chemical universe database GDB-17. *J. Chem. Inf. Model. ***52**(11), 2864–2875. 10.1021/CI300415D/ASSET/IMAGES/CI-2012-00415D_M004.GIF (2012). 23088335 10.1021/ci300415d

[32] Zhang, X. H. et al. Alternation of charge injection and recombination in dye-sensitized solar cells by the addition of nonconjugated bridge to organic dyes. *J. Phys. Chem. C ***117**(5), 2024–2031. 10.1021/JP310425Z/SUPPL_FILE/JP310425Z_SI_001.PDF (2013).

[33] Ørnsø, K. B., Jónsson, E. O., Jacobsen, K. W. & Thygesen, K. S. Importance of the reorganization energy barrier in computational design of porphyrin-based solar cells with cobalt-based redox mediators. *J. Phys. Chem. C ***119**(23), 12792–12800. 10.1021/JP512627E/SUPPL_FILE/JP512627E_SI_001.PDF (2015).

[34] Marcus, R. A. Electron transfer reactions in chemistry. Theory and experiment. *Rev. Mod. Phys. ***65**(3), 599. 10.1103/RevModPhys.65.599 (1993).

[35] Elsenety, M. M. *DSSCs PCE Prediction App. *https://dssc-elsenety.streamlit.app/

