# Supplementary material for: Design and predict the potential of imidazole-based organic dyes in dye-sensitized solar cells using fingerprint machine learning and supported by a web application
Source: Sci Rep. 2024 Nov 3;14:26539. doi: 10.1038/s41598-024-76739-6 (PMC11532345; doi:10.1038/s41598-024-76739-6)
Supplement: Supplementary file 1 — Supplementary Material 1 [file 41598_2024_76739_MOESM1_ESM.docx]

**Supporting Information**

**Design and Predict the Potential of Imidazole-Based Organic Dyes in Dye-Sensitized Solar Cells using Fingerprint Machine Learning and supported by a Web Application**

*Mohamed M. Elsenety*

Department of Chemistry, Faculty of Science, Al-Azhar University, Nasr City, Cairo, 11884, Egypt

**Table S1.** Imidazole structures, and their corresponding reference.

| No. | Reference (DOI) | ANCHORING GROUP | DONOR | Structure |
| --- | --- | --- | --- | --- |
| 1 | 10.1016/j.dyepig.2019.02.045 | cyanoacrylic,carboxylic | imidazole | 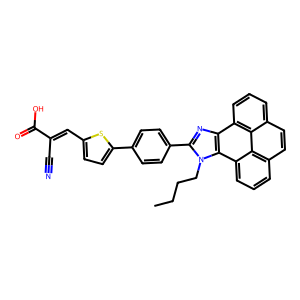 |
| 2 | 10.1016/j.dyepig.2019.02.045 | cyanoacrylic,carboxylic | imidazole | 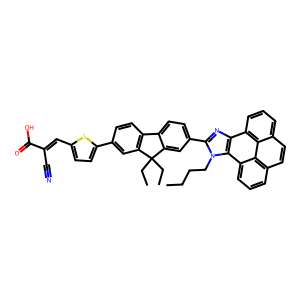 |
| 3 | 10.1016/j.dyepig.2019.02.045 | cyanoacrylic,carboxylic | imidazole | 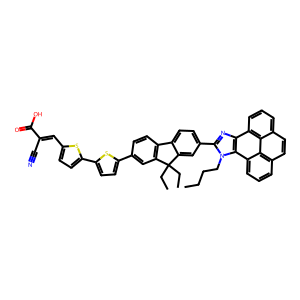 |
| 4 | 10.1016/j.dyepig.2019.02.045 | cyanoacrylic,carboxylic | imidazole | 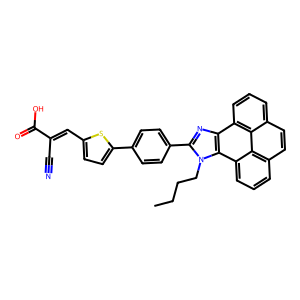 |
| 5 | 10.1039/C6CP06513A | cyanoacrylic,carboxylic | imidazole | 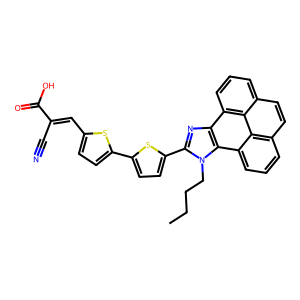 |
| 6 | 10.1039/C6CP06513A | cyanoacrylic,carboxylic | imidazole | 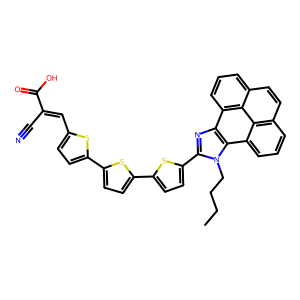 |
| 7 | 10.1039/C6CP06513A | cyanoacrylic,carboxylic | imidazole | 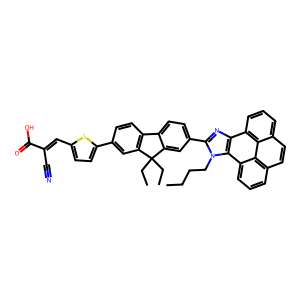 |
| 8 | 10.1039/C6CP06513A | cyanoacrylic,carboxylic | imidazole | 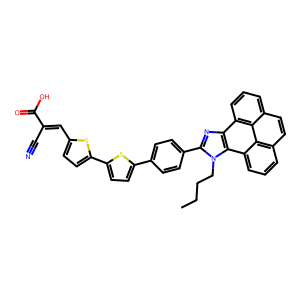 |
| 9 | 10.1039/C6CP06513A | cyanoacrylic,carboxylic | imidazole | 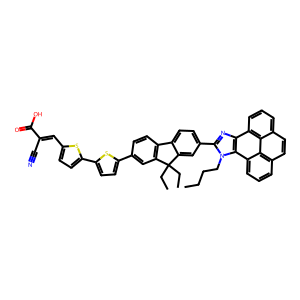 |
| 10 | 10.1021/ol902327p | benzoic,carboxylic | imidazole | 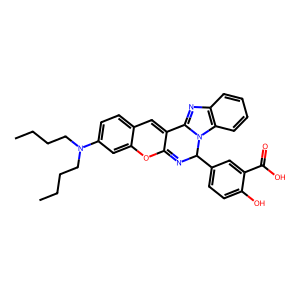 |
| 11 | 10.1039/c3ra43577f | benzoic,carboxylic | imidazole | 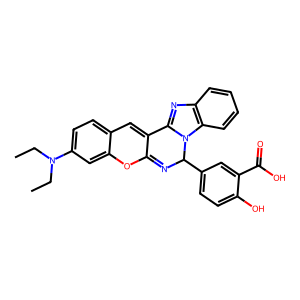 |
| 12 | 10.1039/c3ra43577f | benzoic,carboxylic | imidazole | 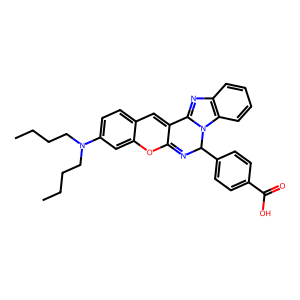 |
| 13 | 10.1016/j.solmat.2004.05.014 | benzoic,carboxylic | imidazole | 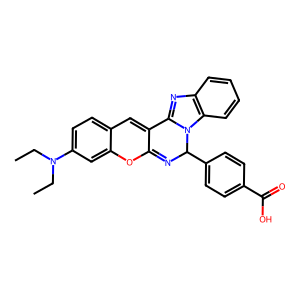 |
| 14 | 10.1002/cssc.201701259 | benzoic,carboxylic | imidazole | 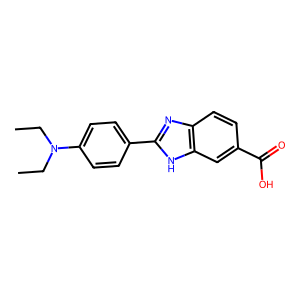 |
| 15 | 10.1002/cssc.201701259 | benzoic,carboxylic | imidazole | 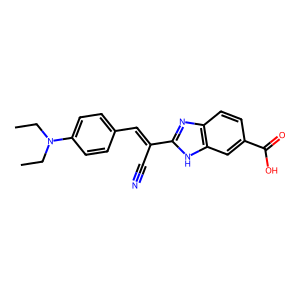 |
| 16 | 10.1002/cssc.201701259 | cyanoacrylic,carboxylic | imidazole | 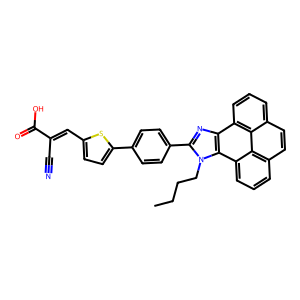 |
| 17 | 10.1002/cssc.201701259 | cyanoacrylic,carboxylic | imidazole | 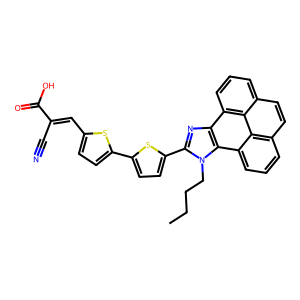 |
| 18 | 10.1039/C9DT02600B | cyanoacrylic,carboxylic | imidazole | 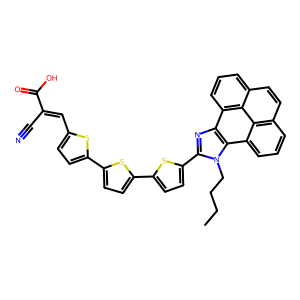 |
| 19 | 10.1016/j.jphotochem.2019.03.043 | cyanoacrylic,carboxylic | imidazole | 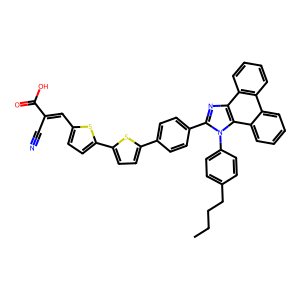 |
| 20 | 10.1016/j.jphotochem.2019.03.043 | cyanoacrylic,carboxylic | imidazole | 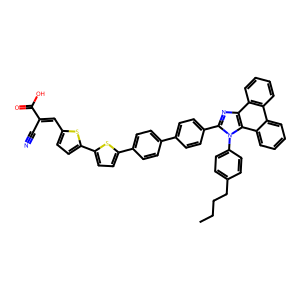 |
| 21 | 10.1016/j.jphotochem.2019.03.043 | cyanoacrylic,carboxylic | imidazole | 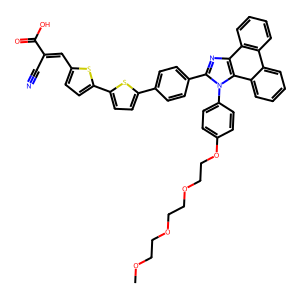 |
| 22 | 10.1016/j.jphotochem.2019.03.043 | cyanoacrylic,carboxylic | imidazole | 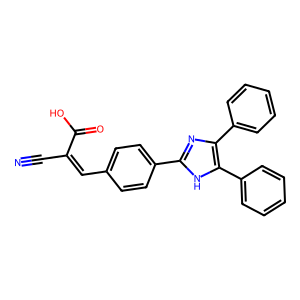 |
| 23 | 10.1016/j.jphotochem.2019.03.043 | carboxylic,rhodanine | imidazole | 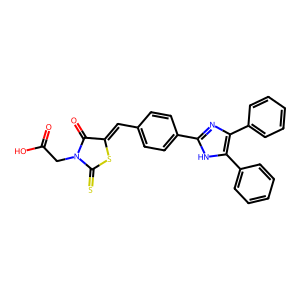 |
| 24 | 10.1016/j.dyepig.2019.02.044 | cyanoacrylic,carboxylic | imidazole | 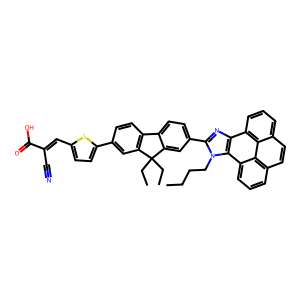 |
| 25 | 10.1016/j.dyepig.2019.02.044 | cyanoacrylic,carboxylic | imidazole | 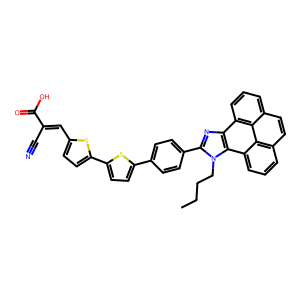 |
| 26 | 10.1016/j.dyepig.2019.02.044 | cyanoacrylic,carboxylic | imidazole | 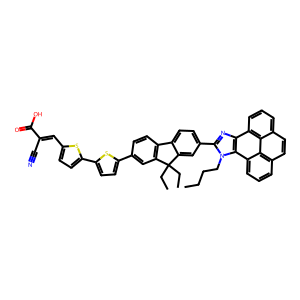 |
| 27 | 10.1016/j.dyepig.2019.02.044 | cyanoacrylic,carboxylic | imidazole | 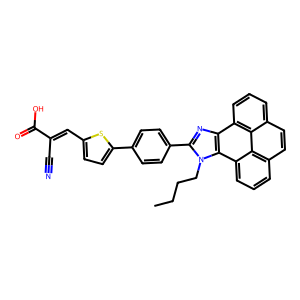 |
| 28 | 10.1016/j.dyepig.2019.02.044 | cyanoacrylic,carboxylic | imidazole | 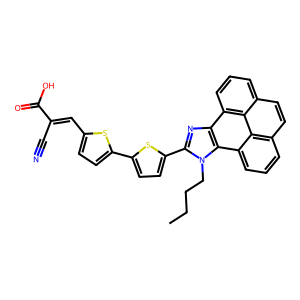 |
| 29 | 10.1016/j.dyepig.2019.02.044 | cyanoacrylic,carboxylic | imidazole | 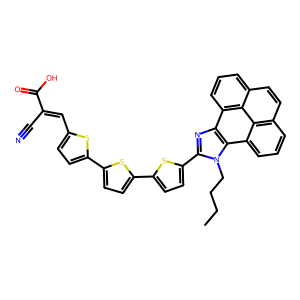 |
| 30 | 10.1021/ol9022936 | cyanoacrylic,carboxylic | imidazole | 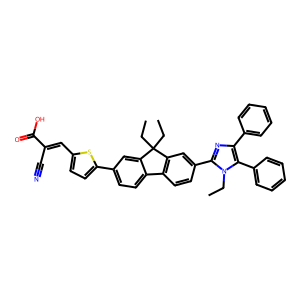 |
| 31 | 10.1021/ol9022936 | cyanoacrylic,carboxylic | imidazole | 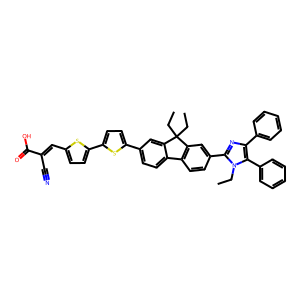 |
| 32 | 10.3390/ijms11010329 | cyanoacrylic,carboxylic | imidazole | 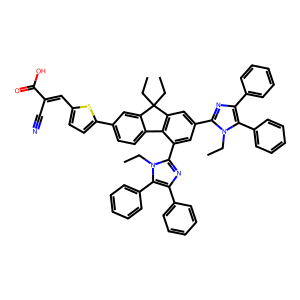 |
| 33 | 10.3390/ijms11010329 | cyanoacrylic,carboxylic | imidazole | 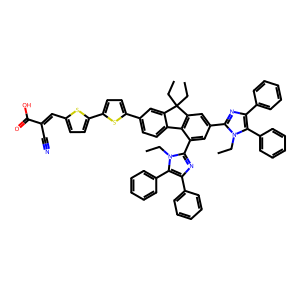 |
| 34 | 10.3390/ijms11010329 | cyanoacrylic,carboxylic | imidazole | 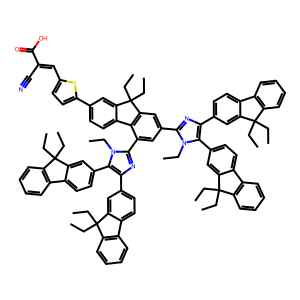 |
| 35 | 10.3390/ijms11010329 | cyanoacrylic,carboxylic | imidazole | 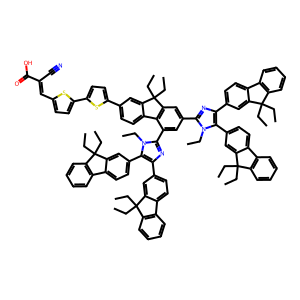 |
| 36 | 10.1039/C6RA17930D | cyanoacrylic,carboxylic | imidazole | 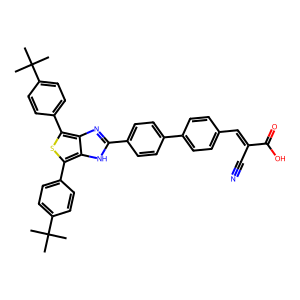 |
| 37 | 10.1039/C6RA17930D | cyanoacrylic,carboxylic | imidazole | 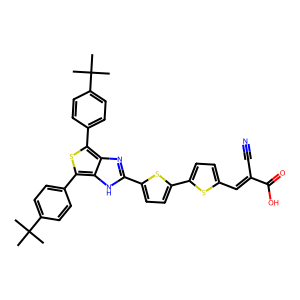 |
| 38 | 10.1039/C6RA17930D | cyanoacrylic,carboxylic | imidazole | 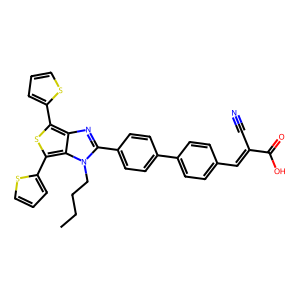 |
| 39 | 10.1039/C2JM34682F | cyanoacrylic,carboxylic | imidazole | 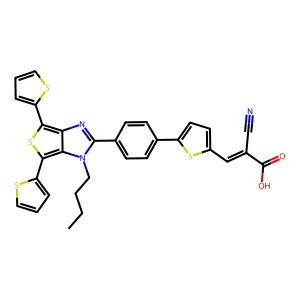 |
| 40 | 10.1039/C2JM34682F | cyanoacrylic,carboxylic | imidazole | 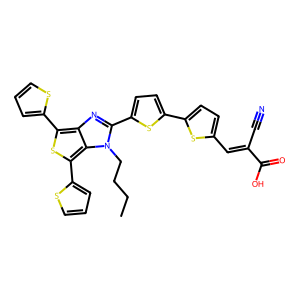 |
| 41 | 10.1039/C2JM34682F | cyanoacrylic,carboxylic | imidazole | 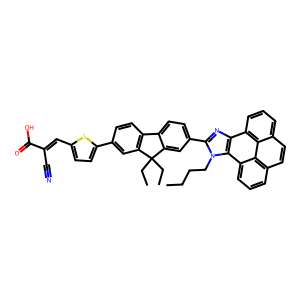 |
| 42 | 10.1039/B203260K | cyanoacrylic,carboxylic | imidazole | 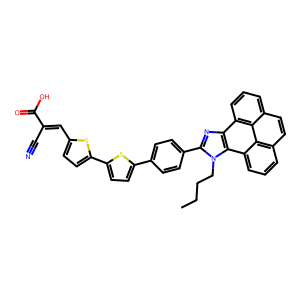 |
| 43 | 10.1039/B203260K | cyanoacrylic,carboxylic | imidazole | 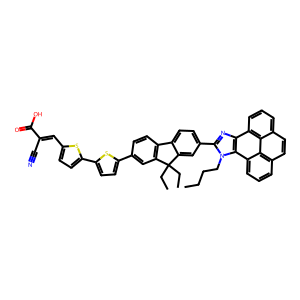 |
| 44 | 10.1039/B203260K | cyanoacrylic,carboxylic | imidazole | 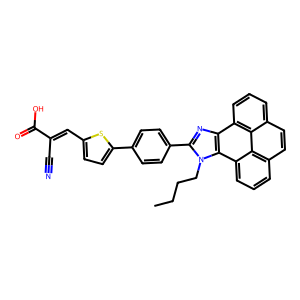 |
| 45 | 10.1039/b603002e | cyanoacrylic,carboxylic | imidazole | 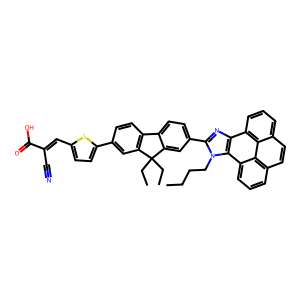 |
| 46 | 10.1021/jp067872t | cyanoacrylic,carboxylic | imidazole | 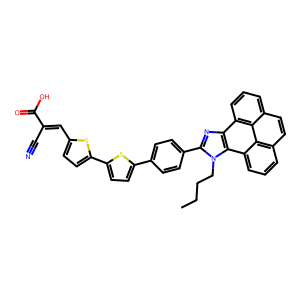 |
| 47 | 10.1016/j.dyepig.2019.02.028 | cyanoacrylic,carboxylic | imidazole | 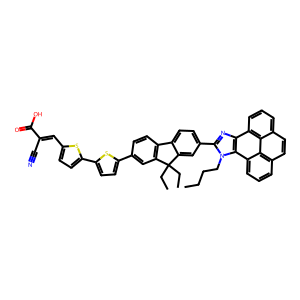 |
| 48 | 10.1021/jp412553z | cyanoacrylic,carboxylic | carbazole,imidazole | 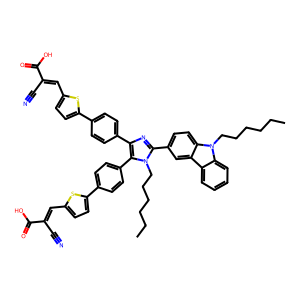 |
| 49 | 10.1021/jp412553z | cyanoacrylic,carboxylic | carbazole,imidazole | 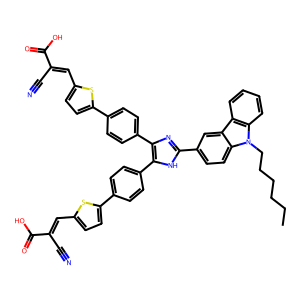 |
| 50 | 10.1021/acsami.6b10162 | cyanoacrylic,carboxylic | indoline,squaraine,imidazole | 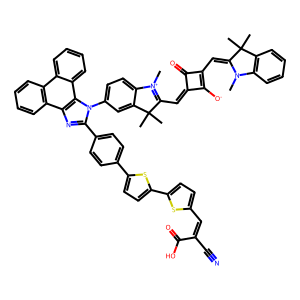 |
| 51 | 10.1002/asia.201200648 | benzoic,carboxylic | perylene,anthracene,imidazole | 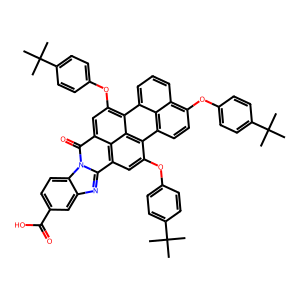 |
| 52 | 10.1002/asia.201200648 | benzoic,carboxylic | perylene,anthracene,imidazole | 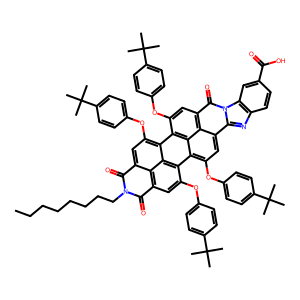 |
| 53 | 10.1002/asia.201200648 | benzoic,carboxylic | perylene,anthracene,imidazole | 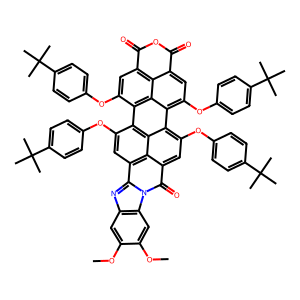 |
| 54 | 10.1002/ajoc.201402214 | carboxylic | perylene,anthracene,imidazole | 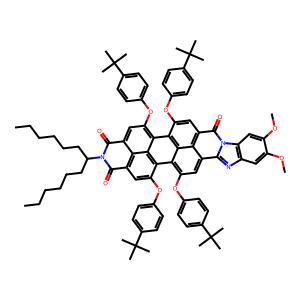 |
| 55 | 10.1039/C8NJ04672G | cyanoacrylic,carboxylic | phenothiazine,imidazole | 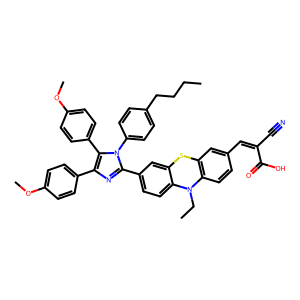 |
| 56 | 10.1039/C5RA07785K | cyanoacrylic,carboxylic | phenothiazine,imidazole | 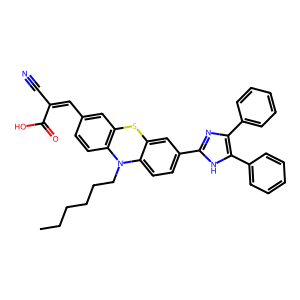 |
| 57 | 10.1039/C5RA07785K | cyanoacrylic,carboxylic | phenothiazine,imidazole | 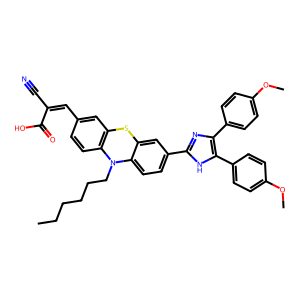 |
| 58 | 10.1039/C5RA07785K | cyanoacrylic,carboxylic | phenothiazine,imidazole | 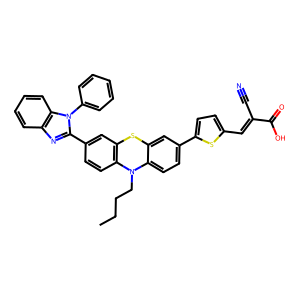 |
| 59 | 10.1039/C3RA22249G | cyanoacrylic,carboxylic | phenothiazine,imidazole | 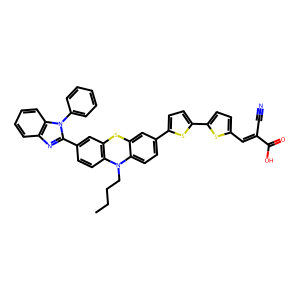 |
| 60 | 10.1039/C3RA22249G | cyanoacrylic,carboxylic | phenothiazine,imidazole | 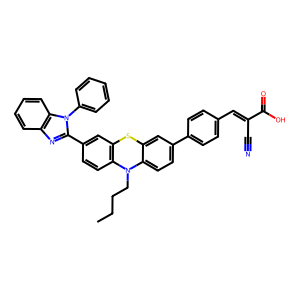 |
| 61 | 10.1002/asia.201100849 | cyanoacrylic,carboxylic | phenothiazine,imidazole | 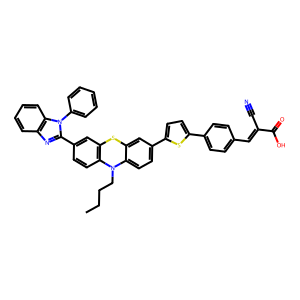 |
| 62 | 10.1002/asia.201100849 | cyanoacrylic,carboxylic | phenothiazine,imidazole | 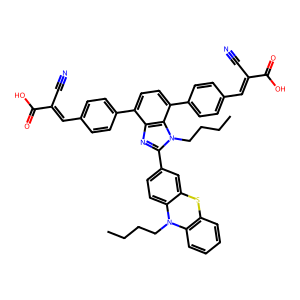 |
| 63 | 10.1002/asia.201100849 | cyanoacrylic,carboxylic | phenothiazine,imidazole | 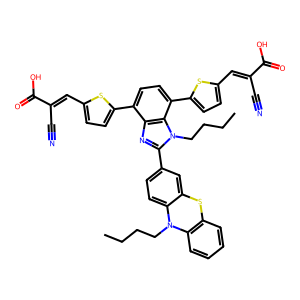 |
| 64 | 10.1002/asia.201100849 | cyanoacrylic,carboxylic | phenothiazine,imidazole | 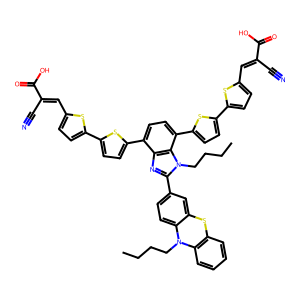 |
| 65 | 10.1039/C8SC05693E | benzoic,carboxylic | tetrahydroquinoline,imidazole | 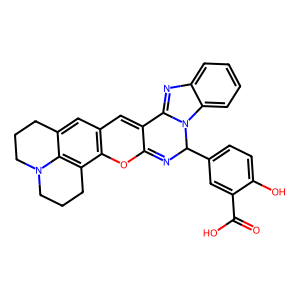 |
| 66 | 10.1039/C8SC05693E | benzoic,carboxylic | tetrahydroquinoline,imidazole | 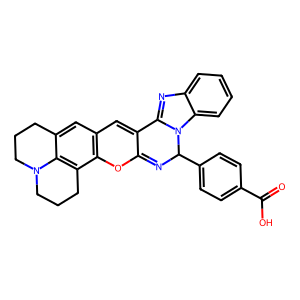 |
| 67 | 10.1016/j.jphotochem.2018.09.033 | cyanoacrylic,carboxylic | triphenylamine,imidazole | 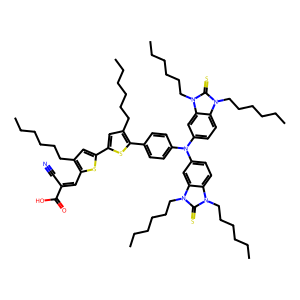 |
| 68 | 10.1016/j.jphotochem.2018.09.033 | cyanoacrylic,carboxylic | triphenylamine,imidazole | 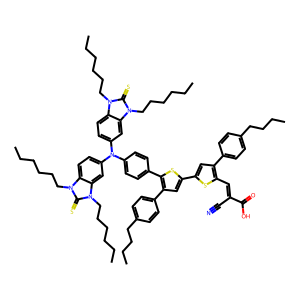 |
| 69 | 10.1002/anie.201808609 | cyanoacrylic,carboxylic | triphenylamine,imidazole | 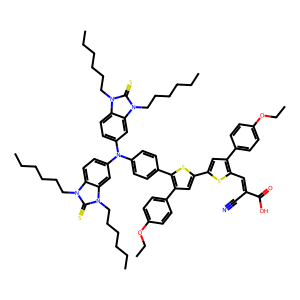 |
| 70 | 10.1002/anie.201808609 | cyanoacrylic,carboxylic | triphenylamine,imidazole | 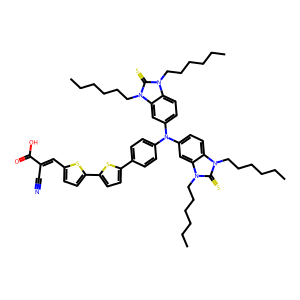 |
| 71 | 10.1002/anie.201808609 | cyanoacrylic,carboxylic | triphenylamine,imidazole | 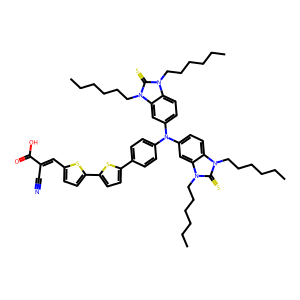 |
| 72 | 10.1002/anie.201808609 | cyanoacrylic,carboxylic | triphenylamine,imidazole | 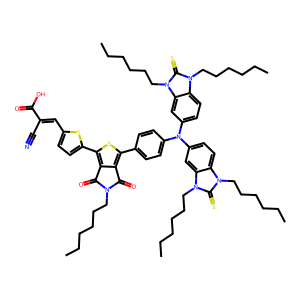 |
| 73 | 10.1021/am404948w | cyanoacrylic,carboxylic | triphenylamine,imidazole | 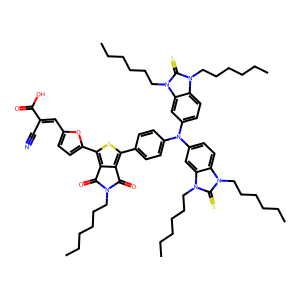 |
| 74 | 10.1021/am404948w | cyanoacrylic,carboxylic | triphenylamine,imidazole | 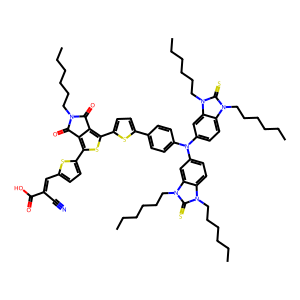 |
| 75 | 10.1021/am404948w | cyanoacrylic,carboxylic | triphenylamine,imidazole | 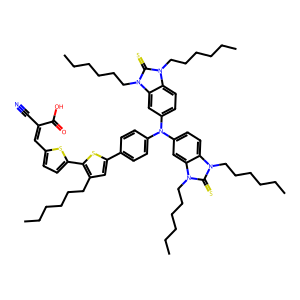 |
| 76 | 10.1021/am404948w | cyanoacrylic,carboxylic | triphenylamine,imidazole | 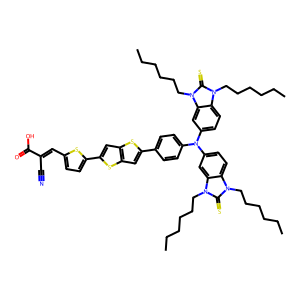 |
| 77 | 10.1021/am404948w | cyanoacrylic,carboxylic | triphenylamine,imidazole | 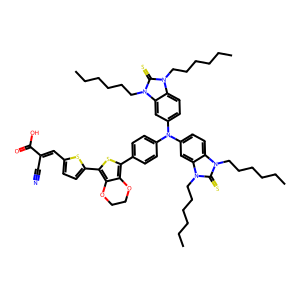 |
| 78 | 10.1039/c3ta11398a | cyanoacrylic,carboxylic | triphenylamine,imidazole | 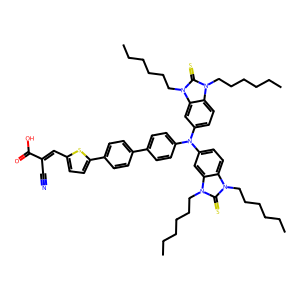 |
| 79 | 10.1039/c3ta11398a | cyanoacrylic,carboxylic | triphenylamine,imidazole | 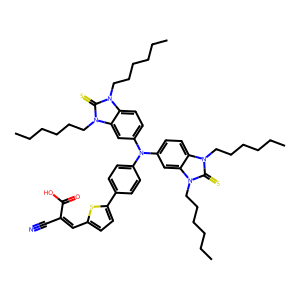 |
| 80 | 10.1016/j.saa.2015.04.074 | cyanoacrylic,carboxylic | triphenylamine,imidazole | 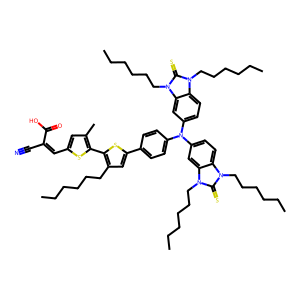 |
| 81 | 10.1016/j.saa.2015.04.074 | cyanoacrylic,carboxylic | triphenylamine,imidazole | 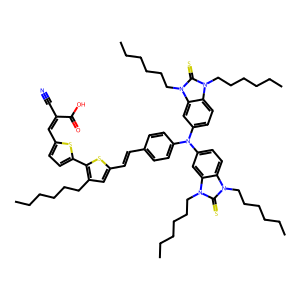 |
| 82 | 10.1016/j.saa.2015.04.074 | cyanoacrylic,carboxylic | triphenylamine,imidazole | 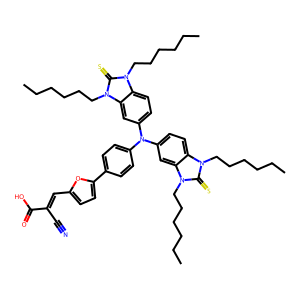 |
| 83 | 10.1002/asia.201700039 | cyanoacrylic,carboxylic | triphenylamine,imidazole | 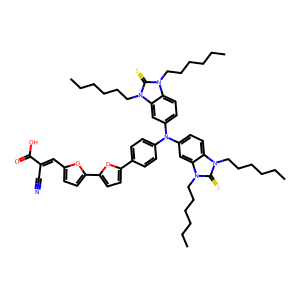 |
| 84 | 10.1002/asia.201700039 | cyanoacrylic,carboxylic | triphenylamine,imidazole | 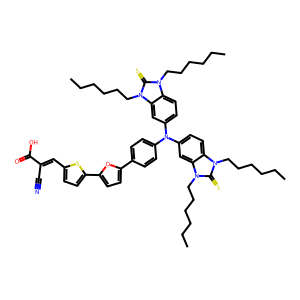 |
| 85 | 10.1002/asia.201700039 | carboxylic | triphenylamine,imidazole | 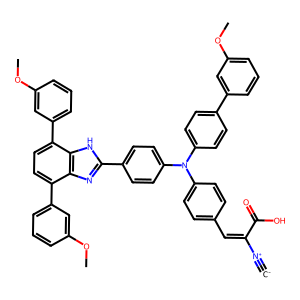 |
| 86 | 10.1002/asia.201700039 | carboxylic | triphenylamine,imidazole | 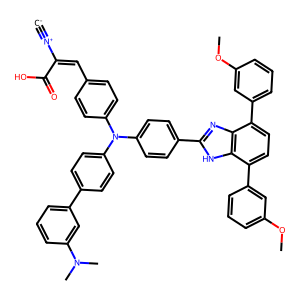 |
| 87 | 10.1002/asia.201700039 | carboxylic | triphenylamine,imidazole | 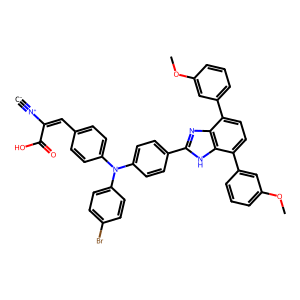 |
| 88 | 10.1002/ajoc.201402214 | carboxylic,rhodanine | triphenylamine,imidazole | 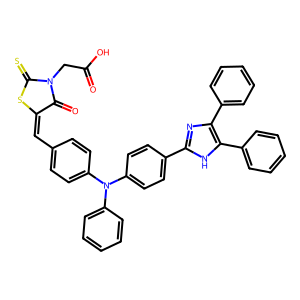 |
| 89 | 10.1002/ajoc.201402214 | cyanoacrylic,carboxylic | triphenylamine,imidazole | 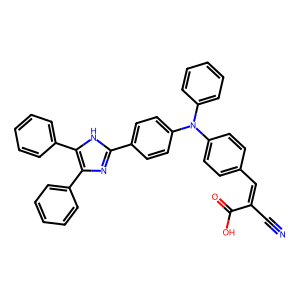 |
| 90 | 10.1002/ajoc.201402214 | benzoic,carboxylic | triphenylamine,imidazole | 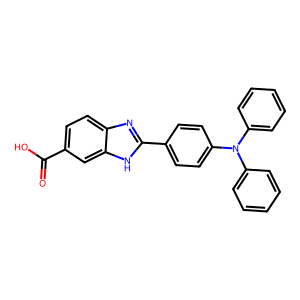 |
| 91 | 10.1021/ol902314z | benzoic,carboxylic | triphenylamine,imidazole | 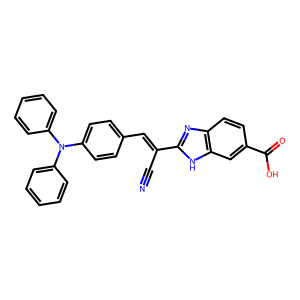 |
| 92 | 10.1039/C5TA09763K | cyanoacrylic,carboxylic | triphenylamine,imidazole | 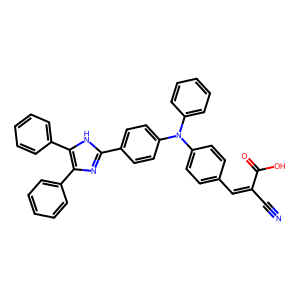 |
| 93 | 10.1039/C5TA09763K | cyanoacrylic,carboxylic | triphenylamine,imidazole | 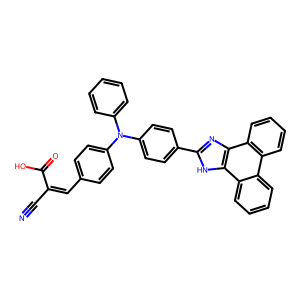 |
| 94 | 10.1039/C5TA09763K | cyanoacrylic,carboxylic | triphenylamine,imidazole | 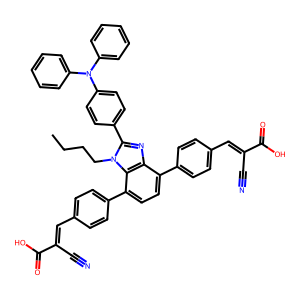 |
| 95 | 10.1039/C5TA09763K | cyanoacrylic,carboxylic | triphenylamine,imidazole | 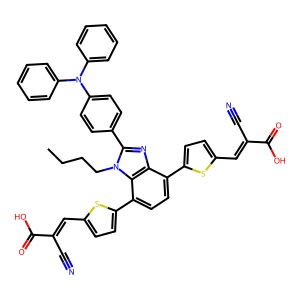 |
| 96 | 10.1039/C5TA09763K | cyanoacrylic,carboxylic | triphenylamine,imidazole | 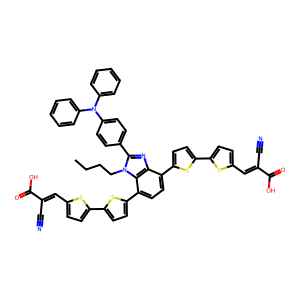 |
| 97 | 10.1039/C4TA04884A | cyanoacrylic,carboxylic | triphenylamine,imidazole | 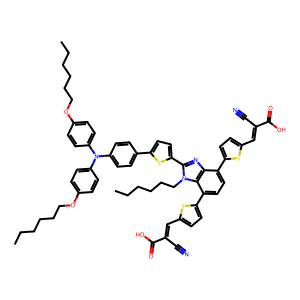 |
| 98 | 10.1039/C4TA04884A | cyanoacrylic,carboxylic | triphenylamine,imidazole | 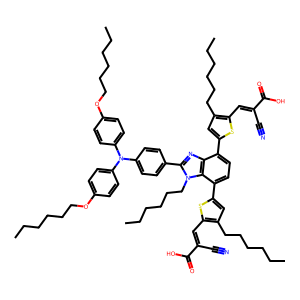 |
| 99 | 10.1039/C4TA04884A | cyanoacrylic,carboxylic | triphenylamine,imidazole | 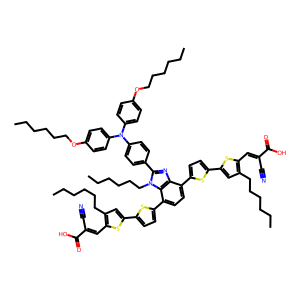 |
| 100 | 10.1039/C2CP40441A | cyanoacrylic,carboxylic | triphenylamine,imidazole | 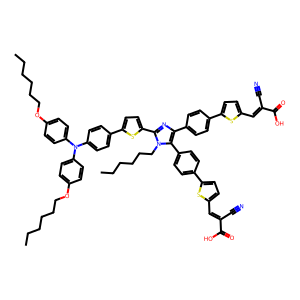 |
| 101 | 10.1039/C2CP40441A | cyanoacrylic,carboxylic | triphenylamine,imidazole |  |
| 102 | 10.1039/C2CP40441A | cyanoacrylic,carboxylic | triphenylamine,imidazole |  |
| 103 | 10.1021/jp310425z | benzoic,carboxylic | triphenylamine,imidazole |  |
| 104 | 10.1021/jp310425z | benzoic,carboxylic | triphenylamine,imidazole |  |
| 105 | 10.1021/jp310425z | cyanoacrylic,carboxylic | triphenylamine,imidazole |  |
| 106 | 10.1016/j.solmat.2006.10.004 | cyanoacrylic,carboxylic | triphenylamine,imidazole |  |
| 107 | 10.1039/C9DT02600B | carboxylic | triphenylamine,imidazole |  |
| 108 | 10.1016/j.dyepig.2019.02.013 | carboxylic | triphenylamine,imidazole |  |
| 109 | 10.1016/j.dyepig.2019.02.013 | carboxylic | triphenylamine,imidazole |  |
| 110 | 10.1016/j.dyepig.2019.02.013 | cyanoacrylic,carboxylic | triphenylamine,imidazole |  |

**Table S2.** 15 different types of the imidazole derivatives structures

| Cpd # | Anchoring_group | Doner__group |
| --- | --- | --- |
| 1 | cyanoacrylic,carboxylic | imidazole |
| 10 | benzoic,carboxylic | imidazole |
| 27 | carboxylic,rhodanine | imidazole |
| 55 | cyanoacrylic,carboxylic | bodipy,imidazole |
| 59 | cyanoacrylic,carboxylic | carbazole,imidazole |
| 61 | cyanoacrylic,carboxylic | indoline,squaraine,imidazole |
| 62 | benzoic,carboxylic | perylene,anthracene,imidazole |
| 65 | carboxylic | perylene,anthracene,imidazole |
| 66 | cyanoacrylic,carboxylic | phenothiazine,imidazole |
| 76 | benzoic,carboxylic | tetrahydroquinoline,imidazole |
| 78 | cyanoacrylic,carboxylic | triphenylamine,imidazole |
| 96 | carboxylic | triphenylamine,imidazole |
| 99 | nitro | triphenylamine,imidazole |
| 100 | carboxylic,rhodanine | triphenylamine,imidazole |
| 102 | benzoic,carboxylic | triphenylamine,imidazole |

**Table S3.** Tanimoto similarity for each two compounds (idx1 and idx2), and the similarity distance between them.

|  | **idx1** | **idx2** | **distance** |
| --- | --- | --- | --- |
| **Cluster 1** | 107 | 109 | 0 |
| **Cluster 2** | 27 | 28 | 0 |
| **Cluster 3** | 24 | 25 | 0 |
| **Cluster 4** | 47 | 48 | 0.017584 |
| **Cluster 5** | 21 | 22 | 0.030602 |
| **Cluster 6** | 82 | 83 | 0.05255 |
| **Cluster 7** | 103 | 104 | 0.058379 |
| **Cluster 8** | 36 | 37 | 0.059324 |
| **Cluster 9** | 19 | 20 | 0.069267 |
| **Cluster 10** | 79 | 81 | 0.079147 |
| **Cluster 11** | 61 | 62 | 0.086915 |
| **Cluster 12** | 92 | 94 | 0.096048 |
| **Cluster 13** | 91 | 93 | 0.097791 |
| **Cluster 14** | 100 | 101 | 0.118965 |
| **Cluster 15** | 35 | 117 | 0.143049 |
| **Cluster 16** | 114 | 118 | 0.159705 |
| **Cluster 17** | 85 | 86 | 0.166204 |
| **Cluster 18** | 70 | 71 | 0.166207 |
| **Cluster 19** | 51 | 52 | 0.18531 |
| **Cluster 20** | 4 | 7 | 0.192323 |
| **Cluster 21** | 6 | 8 | 0.197133 |
| **Cluster 22** | 87 | 89 | 0.201838 |
| **Cluster 23** | 75 | 76 | 0.211006 |
| **Cluster 24** | 66 | 67 | 0.233277 |
| **Cluster 25** | 11 | 54 | 0.235721 |
| **Cluster 26** | 13 | 14 | 0.241345 |
| **Cluster 27** | 102 | 116 | 0.244425 |
| **Cluster 28** | 80 | 119 | 0.246762 |
| **Cluster 29** | 73 | 74 | 0.255656 |
| **Cluster 30** | 77 | 78 | 0.257661 |
| **Cluster 31** | 108 | 110 | 0.273086 |
| **Cluster 32** | 97 | 98 | 0.283315 |
| **Cluster 33** | 18 | 125 | 0.286621 |
| **Cluster 34** | 26 | 111 | 0.29864 |
| **Cluster 35** | 15 | 135 | 0.30419 |
| **Cluster 36** | 2 | 3 | 0.307137 |
| **Cluster 37** | 69 | 127 | 0.323636 |
| **Cluster 38** | 23 | 112 | 0.325245 |
| **Cluster 39** | 64 | 65 | 0.343495 |
| **Cluster 40** | 53 | 88 | 0.344134 |
| **Cluster 41** | 0 | 1 | 0.369102 |
| **Cluster 42** | 55 | 57 | 0.371563 |
| **Cluster 43** | 33 | 34 | 0.372571 |
| **Cluster 44** | 10 | 134 | 0.384802 |
| **Cluster 45** | 95 | 99 | 0.395018 |
| **Cluster 46** | 60 | 120 | 0.402359 |
| **Cluster 47** | 5 | 130 | 0.417658 |
| **Cluster 48** | 41 | 42 | 0.450988 |
| **Cluster 49** | 84 | 115 | 0.461707 |
| **Cluster 50** | 16 | 144 | 0.485436 |
| **Cluster 51** | 56 | 151 | 0.489458 |
| **Cluster 52** | 58 | 59 | 0.521769 |
| **Cluster 53** | 132 | 138 | 0.526423 |
| **Cluster 54** | 139 | 141 | 0.552609 |
| **Cluster 55** | 17 | 106 | 0.590102 |
| **Cluster 56** | 129 | 156 | 0.602606 |
| **Cluster 57** | 63 | 155 | 0.626758 |
| **Cluster 58** | 68 | 146 | 0.66849 |
| **Cluster 59** | 126 | 158 | 0.711894 |
| **Cluster 60** | 123 | 161 | 0.717613 |
| **Cluster 61** | 38 | 40 | 0.722398 |
| **Cluster 62** | 96 | 163 | 0.747198 |
| **Cluster 63** | 29 | 31 | 0.74761 |
| **Cluster 64** | 30 | 72 | 0.76641 |
| **Cluster 65** | 39 | 49 | 0.853819 |
| **Cluster 66** | 121 | 122 | 0.856201 |
| **Cluster 67** | 12 | 46 | 0.875085 |
| **Cluster 68** | 50 | 128 | 0.921088 |
| **Cluster 69** | 154 | 169 | 0.960325 |
| **Cluster 70** | 105 | 148 | 0.978225 |
| **Cluster 71** | 9 | 136 | 0.978601 |
| **Cluster 72** | 45 | 153 | 0.995502 |
| **Cluster 73** | 32 | 44 | 1.054667 |
| **Cluster 74** | 90 | 170 | 1.064485 |
| **Cluster 75** | 131 | 149 | 1.071428 |
| **Cluster 76** | 145 | 150 | 1.124872 |
| **Cluster 77** | 168 | 171 | 1.127853 |
| **Cluster 78** | 43 | 157 | 1.146094 |
| **Cluster 79** | 176 | 179 | 1.241457 |
| **Cluster 80** | 160 | 164 | 1.271736 |
| **Cluster 81** | 133 | 182 | 1.276593 |
| **Cluster 82** | 143 | 147 | 1.277792 |
| **Cluster 83** | 178 | 181 | 1.290076 |
| **Cluster 84** | 113 | 177 | 1.339908 |
| **Cluster 85** | 172 | 173 | 1.431837 |
| **Cluster 86** | 159 | 192 | 1.501467 |
| **Cluster 87** | 140 | 184 | 1.51402 |
| **Cluster 88** | 152 | 183 | 1.563976 |
| **Cluster 89** | 189 | 193 | 1.579163 |
| **Cluster 90** | 190 | 197 | 1.810328 |
| **Cluster 91** | 180 | 196 | 1.814506 |
| **Cluster 92** | 174 | 175 | 1.897195 |
| **Cluster 93** | 167 | 195 | 1.913865 |
| **Cluster 94** | 124 | 188 | 1.94395 |
| **Cluster 95** | 162 | 200 | 2.176566 |
| **Cluster 96** | 198 | 202 | 2.336909 |
| **Cluster 97** | 137 | 201 | 2.364113 |
| **Cluster 98** | 185 | 203 | 2.508893 |
| **Cluster 99** | 194 | 199 | 2.581463 |
| **Cluster 100** | 186 | 205 | 2.80214 |
| **Cluster 101** | 166 | 208 | 2.883464 |
| **Cluster 102** | 187 | 210 | 3.011998 |
| **Cluster 103** | 204 | 209 | 3.061521 |
| **Cluster 104** | 142 | 207 | 3.480869 |
| **Cluster 105** | 191 | 206 | 3.864121 |
| **Cluster 106** | 165 | 214 | 4.360202 |
| **Cluster 107** | 213 | 215 | 5.551423 |
| **Cluster 108** | 211 | 216 | 6.519192 |
| **Cluster 109** | 212 | 217 | 11.97642 |

**Table S4.** DayLight Fingerprint_models_Score

| **Model** | **CV** | **R2 Score** | **Std**  **r2_score** | **MAE** | **std_mae** | **MSE** | **std_mse** |
| --- | --- | --- | --- | --- | --- | --- | --- |
| RandomForestRegressor | 3 | 0.405 | 0.094 | 1.412 | 0.050 | 3.123 | 0.165 |
| GradientBoostingRegressor | 3 | 0.393 | 0.156 | 1.398 | 0.049 | 3.124 | 0.177 |
| ExtraTreesRegressor | 3 | 0.127 | 0.172 | 1.583 | 0.087 | 4.586 | 0.545 |
| AdaBoostRegressor | 3 | 0.401 | 0.182 | 1.377 | 0.242 | 3.084 | 0.716 |
| BaggingRegressor | 3 | 0.379 | 0.086 | 1.457 | 0.053 | 3.277 | 0.281 |
| SVR | 3 | 0.254 | 0.120 | 1.631 | 0.140 | 3.932 | 0.449 |
| KNeighborsRegressor | 3 | 0.263 | 0.028 | 1.633 | 0.118 | 4.005 | 0.865 |
| MLPRegressor | 3 | 0.420 | 0.203 | 1.368 | 0.184 | 2.928 | 0.541 |
| XGBRegressor | 3 | 0.331 | 0.311 | 1.401 | 0.170 | 3.291 | 0.924 |
| LGBMRegressor | 3 | 0.463 | 0.123 | 1.343 | 0.023 | 2.791 | 0.180 |
| CatBoostRegressor | 3 | 0.413 | 0.132 | 1.410 | 0.145 | 3.057 | 0.415 |
| DecisionTreeRegressor | 3 | -0.059 | 0.158 | 1.709 | 0.028 | 5.595 | 0.568 |
| RandomForestRegressor | 5 | 0.519 | 0.083 | 1.206 | 0.037 | 2.475 | 0.258 |
| GradientBoostingRegressor | 5 | 0.483 | 0.183 | 1.197 | 0.139 | 2.615 | 0.524 |
| ExtraTreesRegressor | 5 | 0.280 | 0.150 | 1.370 | 0.213 | 3.787 | 1.008 |
| AdaBoostRegressor | 5 | 0.540 | 0.070 | 1.188 | 0.064 | 2.374 | 0.206 |
| BaggingRegressor | 5 | 0.377 | 0.238 | 1.400 | 0.137 | 3.120 | 0.729 |
| SVR | 5 | 0.342 | 0.088 | 1.537 | 0.153 | 3.443 | 0.579 |
| KNeighborsRegressor | 5 | 0.417 | 0.124 | 1.395 | 0.102 | 3.014 | 0.644 |
| MLPRegressor | 5 | 0.479 | 0.206 | 1.295 | 0.146 | 2.592 | 0.540 |
| XGBRegressor | 5 | 0.493 | 0.124 | 1.215 | 0.135 | 2.607 | 0.402 |
| LGBMRegressor | 5 | 0.536 | 0.147 | 1.212 | 0.101 | 2.340 | 0.553 |
| CatBoostRegressor | 5 | 0.493 | 0.103 | 1.262 | 0.095 | 2.609 | 0.347 |
| DecisionTreeRegressor | 5 | 0.157 | 0.402 | 1.488 | 0.381 | 4.285 | 1.632 |
| RandomForestRegressor | 7 | 0.527 | 0.123 | 1.186 | 0.147 | 2.409 | 0.687 |
| GradientBoostingRegressor | 7 | 0.530 | 0.171 | 1.109 | 0.179 | 2.358 | 0.781 |
| ExtraTreesRegressor | 7 | 0.265 | 0.300 | 1.337 | 0.272 | 3.565 | 1.153 |
| AdaBoostRegressor | 7 | 0.578 | 0.088 | 1.113 | 0.077 | 2.154 | 0.437 |
| BaggingRegressor | 7 | 0.543 | 0.069 | 1.200 | 0.198 | 2.427 | 0.734 |
| SVR | 7 | 0.399 | 0.131 | 1.445 | 0.174 | 3.081 | 0.591 |
| KNeighborsRegressor | 7 | 0.453 | 0.143 | 1.330 | 0.171 | 2.868 | 0.896 |
| MLPRegressor | 7 | 0.612 | 0.164 | 1.174 | 0.240 | 2.258 | 0.852 |
| XGBRegressor | 7 | 0.419 | 0.214 | 1.216 | 0.197 | 2.841 | 0.715 |
| LGBMRegressor | 7 | 0.576 | 0.127 | 1.141 | 0.133 | 2.116 | 0.438 |
| CatBoostRegressor | 7 | 0.562 | 0.120 | 1.132 | 0.189 | 2.234 | 0.548 |
| DecisionTreeRegressor | 7 | 0.283 | 0.243 | 1.368 | 0.307 | 3.600 | 1.300 |
| RandomForestRegressor | 10 | 0.520 | 0.169 | 1.155 | 0.177 | 2.290 | 0.866 |
| GradientBoostingRegressor | 10 | 0.481 | 0.258 | 1.116 | 0.241 | 2.389 | 1.138 |
| ExtraTreesRegressor | 10 | 0.321 | 0.376 | 1.230 | 0.337 | 3.341 | 2.057 |
| AdaBoostRegressor | 10 | 0.519 | 0.168 | 1.161 | 0.128 | 2.243 | 0.575 |
| BaggingRegressor | 10 | 0.446 | 0.193 | 1.269 | 0.238 | 2.682 | 1.060 |
| SVR | 10 | 0.351 | 0.192 | 1.468 | 0.207 | 3.190 | 0.957 |
| KNeighborsRegressor | 10 | 0.446 | 0.210 | 1.262 | 0.149 | 2.670 | 0.841 |
| MLPRegressor | 10 | 0.472 | 0.295 | 1.229 | 0.321 | 2.424 | 1.119 |
| XGBRegressor | 10 | 0.436 | 0.216 | 1.167 | 0.226 | 2.675 | 1.073 |
| LGBMRegressor | 10 | 0.529 | 0.201 | 1.137 | 0.189 | 2.134 | 0.551 |
| CatBoostRegressor | 10 | 0.486 | 0.256 | 1.162 | 0.286 | 2.371 | 0.942 |
| DecisionTreeRegressor | 10 | 0.152 | 0.618 | 1.360 | 0.452 | 4.192 | 3.331 |

**Table S5.** Top 238 chemical structures with their predicted PCE% over one million smile structures.

| **SMILES** | **Predicted_PCE %** |
| --- | --- |
| CC1CCN2C1COC1=C(N)NC(C(O)=O)=C21 | 7.53 |
| CC1C(C#N)N2C3=C(N=C(O)C2=N)C(O)CN13 | 7.09 |
| CC1=CC(=O)C2=C3C(OC=N2)C2=C(OC=C2)N13 | 7.65 |
| CC1=CC(=O)C2=C(COCC3=C(N2)N=NO3)N1 | 7.39 |
| CC1=CC(=O)C2OC2C2=C1N=C(N2)S(O)(=O)=O | 7.29 |
| CC1=C(CO)SC2=C1C1COC(=O)C2NC=N1 | 7.29 |
| CC1=C(F)C2=C(C(O)C3=C2N=C(N)N3)C(N)=N1 | 7.15 |
| CC1CN1C1=C(CO)NC2=C1ON=C2C#C | 7.13 |
| CC1CN2C3=C4C(NC5C(=O)N1C2=C45)C(=N)N3 | 7.07 |
| CC1=C(N2CCC2)C(=O)NC2=C1ON=C2O | 7.82 |
| CC1CN2C=CC(=O)C3=C2C(O1)C(N3)C(O)=O | 7.04 |
| CC1=CN2C(=N1)C(O)C1=NC(N)=C(C)C(F)=C21 | 7.78 |
| CC1=CNC2=C1N1C(=CC(C)=C(N)C1=N)C2O | 7.19 |
| CC1=C(N)C2=C(S1)C1CN1C1=NON=C1O2 | 7.23 |
| CC1=C(N)C(=N)NC2=C1C1CCC(O1)OC2=O | 7.60 |
| CC1=C(N)NC2=C1N1CC(=O)C(O)CC1C2O | 7.15 |
| CC1=C(N)N=C2C(O)C3=C(N12)C(=O)NC(N)=N3 | 7.10 |
| CC1=C(N)N=C2C(O)C3=NC(F)=CC(C)=C3N12 | 7.36 |
| CC1=C(N)N=CC=C2OCOC2=C(N1)C(O)=O | 7.29 |
| CC1=C(N)SC2=C1N1C(=NC(Cl)=CC1=O)C2O | 7.45 |
| CC1C(O)C2=C3N1C(CC#N)CC3=CC(=N)N2 | 7.27 |
| CC1C(O)C2=C3N1CCN3C(NCCO)=C2 | 7.01 |
| CC1C(O)C2=CC3=C(N12)N1N=NC=C1C3 | 7.28 |
| CC1=C(O)C2=C(COCC3=C(C2)N=C(N)N3)S1 | 7.12 |
| CC1C(O)C2=C(N1C)C1=C(N2)C(N)=NC(=O)S1 | 8.36 |
| CC1COC2=C(N)C3=C(N12)C(C)=C(N3)C(O)=O | 7.60 |
| CC1COC2=C(N)NC3=C2N1C(=N)C(N)=C3O | 7.37 |
| CC1=COC(=C1)C1=C(N)C(O)=NC2=NON=C12 | 7.01 |
| CC1C(O)CC(O)C2=C(C=C(Br)C(=N)N2)N1C | 7.03 |
| CC1=C(O)C(F)=C2N=C(O)C(NCC=O)=C2S1 | 7.12 |
| CC1C(OC=O)C2=C(N)C(=N)OC3=C2N1N=N3 | 7.02 |
| CC1=C(O)N=C2C(O)C3=C(N12)C(F)=NC(=O)N3 | 7.23 |
| CC1=C(O)N=C2C(O)C3=C(N12)C(N)=NC(=O)N3 | 7.26 |
| CC1=CSC2=C1C(O)C1=C2C(Br)=C(N)C(=O)N1 | 7.69 |
| CC1N2C3=C(N=CC2=N)C(O)C2=NC(C)=C1N32 | 7.08 |
| CC1N2C(C3=C1OC(=N)S3)=C(O)C(N)=NC2=O | 7.05 |
| CC1N2C(=CC3=C2NCC3O)C2=C1C=CN2C | 7.25 |
| CC1N2C=CN=C2C2=C(NC(=O)N=C12)C(O)=O | 7.15 |
| CC1N2C(CO)=NC3=C2N2C1=CC(C)=C2C3O | 7.24 |
| CC1N2C(CO)=NC(C)=C2C2=NC(N)=CC=C12 | 7.02 |
| CC1N2N=C(N)C(O)=C2C2=NC(=N)C=CC=C12 | 7.81 |
| CC1N2N=NC=C2C2=CC(O)=CC(=O)C(N)=C12 | 7.07 |
| CC1N2N=NC(O)=C2OC2=CSC(CO)=C12 | 7.01 |
| CC1=NC2=C3C(SC4=C(CO)NC(C2=O)=C34)=C1 | 7.55 |
| CC1NC2CC1NC1=C(NC(=O)N=C1O)C2=O | 7.03 |
| CC1=NC2=C(C=C1)C1=C(N2)C(O)C2=C1OC=N2 | 7.01 |
| CC1=NC2=C(C=C1N)C1=C(CO)NC(N)=C1O2 | 7.05 |
| CC1NC2=C(C=C3CNC1C3O)C(N)=NO2 | 7.04 |
| CC1=NC2=C(N1CC#N)C(F)=C(CO)NC2=O | 7.08 |
| CC1=NC2=C(N1)N1CC2OC2=C1C(N)=NO2 | 8.53 |
| CC1NC2CNC3=C2C1=C(NC3=O)C(O)C#N | 7.97 |
| CC1=NC2=C(NC3=NC=NN3)C(O)=CC2=NO1 | 7.20 |
| CC1NC2=C(NC(N)=C2O)C2=C1ON=C2CO | 7.41 |
| CC1NC2=C(ON=C2O)C2=NOC(=N)C(Br)=C12 | 7.25 |
| C1C2N=C3OC4C5=C6N(N=C5)C1C1=C2C3=C4N61 | 7.33 |
| CC1=NC=C2N3C=C(O)C(NC=N)=C3CC2=C1 | 7.08 |
| CC1N(CC#N)C2=C(NC(O)=C2C1=O)C(O)=O | 7.13 |
| CC1=NC(CO)=C2OC3=C(N12)C(=N)ON=C3N | 7.21 |
| CC1=NC(CO)=NC2=C(NCC(O)=O)N=NN12 | 7.15 |
| CC1=NC(NC2=C(CO)NC(=O)C(N)=C2)=NO1 | 7.45 |
| CC12CN3CC(O)C4=C3C1=C(N4)C1=NOC=C21 | 7.06 |
| CC1=NOC2=C1C(O)C1=C2C(F)=C(N)C(=O)S1 | 7.02 |
| CC1OC2C3=C1N=CN3C1=C2C(=O)OC=C1Cl | 7.40 |
| CC1OC2C3=C(NC4=C2C1=C(CO)N4)N=CN3 | 7.33 |
| CC1OC2C3=C(N=CN3)N3C2=C(O1)C=CC3=N | 7.81 |
| CC1OC2C3=C(N=C(N)O3)N3C(CO)=NC1=C23 | 7.08 |
| CC1OC2C3=C(N=NN3)N3C(CO)=NC1=C23 | 7.97 |
| CC1OC2=CC(=O)C3=C4N2C1CC4=C(CO)N3 | 7.49 |
| CC1OC2CN3C2C(O1)C1=C3N=C(CO)N1 | 7.21 |
| CC1OC2=C(ON=C2N)C2=C1C=NC(=O)N2 | 7.18 |
| CC1OC=NC2=C(O)C(NC3=C1NC=N3)=NO2 | 7.06 |
| C1CC2=C3N1C1CC4=C5C1N3C1=C2OC(=C4)N51 | 7.05 |
| CCC1=C2C(C)OC3C(=N)NC(N1CCO)=C23 | 7.10 |
| CCC1=C2CN3N=C(NC)C4=C3C2=C(CO4)S1 | 7.31 |
| CCC1=C2C(O)C3=C(OC(=O)N3)N2C(=N)C=C1 | 7.09 |
| CCC1=C2N3N=C(N)N=C3OC2=C(N1)C(O)=O | 8.02 |
| CCC1=C2N(C(C)=C1O)C1=C(N=NO1)C2=O | 7.37 |
| CCC1=CC2=C3N1C1CCC4=C1N3C(=C4)C2O | 7.58 |
| CCC1=CC2=C3N1CCOC1=CN=C(C2O)N31 | 7.07 |
| CCC1CC2=C(N1C)C1=C(N2)C(O)=NN1C=O | 7.02 |
| CCC1CC2NC(=O)C3=C2C(N1C)=C(CO)N3 | 7.20 |
| CCC1CCC2=C(NC3=C2ON=C3O)N1CC | 7.15 |
| CCC1=C(CC2=C(NC(N)=N2)C(O)=O)ON=N1 | 7.16 |
| CCC1=C(CO)NC(F)=C2C1=NC(N)=C2C#C | 7.13 |
| CCC1=CN2C(=N1)C(O)C1=C2C(Br)=CC(=O)S1 | 7.68 |
| CCC1=CNC2=C1C(=O)C1=C(C(O)CN1)N2C | 7.11 |
| CCC1=C(N(C)C=O)C2=C(NC1=O)C(N)=NO2 | 7.41 |
| CCC1=C(NC=O)SC2=C1C1=C(N=NO1)C2O | 7.29 |
| CCC1=C(N)NC2=C1N(C(C#N)C2O)C(C)=O | 7.37 |
| CCC1=COC2=C1N1C(=NC(Br)=NC1=O)C2O | 7.18 |
| CCC1NC2=C3COC4CN5C(C)C1N2C5=C34 | 8.43 |
| C#CCC1=C2NC3=NON=C3OC2=C(S1)C#N | 7.04 |
| CCCC1=C2NC(C)C3=C(NC(N1)=C23)C(O)=O | 7.16 |
| CC12OC(CN=C(CO)O1)C1=C2N=C(S1)C#N | 7.01 |
| BrC1=CC2=C(N3CC2OC2=CON=C32)C(=O)N1 | 8.34 |
| C1NC2=C3C1OC1OCC4=NNC(C3=NN2)=C14 | 7.19 |
| C1NC2=C3C1OC=NC1=C4N(CC(N2)=C34)C=N1 | 7.27 |
| CCC(C)(C)N1C=NC2=C(N)NC(C(O)=O)=C12 | 7.57 |
| CC1=C2CC3=C4N5C(=N3)C(O)C3=C5C(N24)=C1O3 | 7.51 |
| CC1=C2CC3=C(NC(N)=N3)C(=O)OCC2=NO1 | 7.35 |
| C1OC2=C3C1NC1CC4OC5=NN=C2N5C4=C31 | 7.09 |
| CC(CC=O)N1CC(=O)C2=C(N)NC(CO)=C12 | 7.28 |
| CC(C)N1CC2OC3=CC=C(O)C4=NC1=C2N34 | 8.11 |
| CCCN1C(N)=CC2=CC(=N)NC(C(O)=O)=C12 | 7.05 |
| CC(C)N1N=NC2=C1CC1=C(N)C(O)=C(C)N21 | 7.22 |
| CC(C)N1N=NC=C1OC1=C(N)NC(CO)=C1 | 7.05 |
| CC(C)NC1=NC2=C(C=CNC2=C1O)C(C)O | 7.39 |
| CC(C)N(C)C1=C2NC(O)=NC(N)=C2N=C1O | 7.04 |
| CC(C)N(C)C1=C(CO)C2=CON=C2N=C1O | 7.02 |
| CCCOC1C2=C(N=C(N)N2)C2=NOC(N)=C12 | 7.42 |
| CCCOC1=C2NC(C(O)=O)=C(N)N=C2N=C1 | 7.36 |
| CC(C)OC1=C2NC(=O)NC(C(O)=O)=C2N=C1 | 7.28 |
| CCC(O)C1C2OC(=O)CN1C1=C2N=C(N)N1 | 7.19 |
| CCCOC1=NC2=NOC(N)=C2C=C1N(C)C | 7.13 |
| CC1C2CCC(O)C3=C(N12)C(=O)C=C(C)N3 | 7.27 |
| CCN1C2=C3N4C(CC3=CC1=N)=CN=C4C2O | 7.33 |
| CCN1C2=C3N(CC2O)CC2=NNC(=C32)C1=N | 7.00 |
| CCN1C2=CC=C3CC4=C(N23)C1=C(O)C(=O)N4 | 7.65 |
| CCN1C2=C(C=CO2)C(=O)OC2=C1C(N)=NO2 | 7.23 |
| CCN1C2=C(NC(=C2)C(O)=O)C(C)=C1N(C)C | 7.16 |
| CCN1C2C(=NO)C(O)C=C2C2=C1C(N)=NS2 | 7.01 |
| CCN1CC2NC2CC2=C(N)NC(CO)=C12 | 7.25 |
| CCN1CCC2=C(NC(OCC=O)=C12)C(O)=O | 7.69 |
| CCN1C(C)CC2C1C1=C(NC(CO)=N1)N2C | 7.52 |
| CCN1CCC(=N)OC2=C1C(=O)NC(CO)=C2 | 7.23 |
| CCN1C(C#N)C(O)C2=C(N)C(=O)NC(F)=C12 | 7.08 |
| CCN1C(CO)=C2C3=CN=CN3C3CNC1=C23 | 7.21 |
| CCN1C=NC2=C1N1C(=NC(Br)=CC1=O)C2O | 7.07 |
| CCN1C(N)=NC2=C(NC(C(O)=O)=C12)N=CN | 7.34 |
| CCN1N=CC2=C1NC1=C2NCC(=O)CC1O | 7.57 |
| CCNC1=C2N=C(NC)C(O)=C2NC(F)=C1C | 7.01 |
| CC(NC1=CC2=C(NC1=N)C(O)CN2C)C#N | 7.13 |
| CCNC1=C(O)N=C2COC3CNC(=C23)C1=O | 7.15 |
| CC(NC1=NC2=C(N1)C1=NOC=C1C2O)C#C | 7.15 |
| CCN=C1NC(CO)=C(C=O)C2=C1ON=C2N | 7.05 |
| CC(N)C1OC2C3=C(N4N=NC1=C24)C(O)=NN3 | 7.02 |
| CCN=C1ON=C(N)C2=C1C1CCC(CO2)O1 | 7.17 |
| CCN(C)C1=C2N=C(N)C(O)=C2OC(C)=C1O | 7.15 |
| CCN(C)C1=CC=C(NC)N2N=NC(CO)=C12 | 7.49 |
| CCN(C)C1=CC=C(O)N2C1=CC(N)=C2CO | 7.29 |
| CCN(C)C1=C(CO)NC(=O)C2=C1C(N)=CN2 | 7.11 |
| CCN(C)C1=C(F)C2=C(NC=C2N=C1)C(C)O | 7.15 |
| CCN(C)C1=C(NC2=C1C=CNC2=O)C(O)=O | 7.70 |
| CCN(C)C1=COC2=C1NC(=C2O)S(O)(=O)=O | 7.08 |
| CCN(CC)C1=CC2=C(NC3=NON=C3O2)S1 | 7.16 |
| CCN(CC#C)C1=NOC2=C1NC(C)=C2NC | 7.20 |
| CCN(CC=O)C1=C(NC(N)=C1O)C(=O)OC | 7.11 |
| CC(=NO)C1OC2C3=C(N=NN3)C3=NOC1=C23 | 7.10 |
| CC(O)C1=C2C3=C(SC2=C(N)C(=N)N1)N=NO3 | 7.55 |
| CCOC1=C2C(=NO1)N=C(NC=O)C(O)=C2Br | 7.16 |
| CC(=O)C1=C2C(OCCNC3=C2ON=N3)=CN1 | 7.10 |
| CC(O)C1=C2N3C(OC2=CN1)=C(Br)C=CC3=N | 7.10 |
| CC(O)C1=C2NCC3N2C(=C1O)C1=C3N=NO1 | 7.07 |
| CC(=O)C1=C2N=C(N)C3=C(C(O)=CO3)C2=CN1 | 7.58 |
| CC(=O)C1=C2NC(=N)C(N)=C2NC(CO)=C1F | 7.19 |
| CC(=O)C1=C2N=C(N)C(O)=C2NC(C)=C1CO | 7.59 |
| CC(=O)C1=C2N=C(N)C(O)=C(F)N2N=C1C#N | 7.07 |
| CC(O)C1=CC2=C(N1)C1CN2C2=C(O1)C=CO2 | 7.49 |
| CC(O)C1=CC2=C(N1)C1=C(NC2C)C(N)=NO1 | 7.28 |
| CC(O)C1=CC2=C(N1)N1CC(=O)OC2C1C#C | 7.72 |
| CC(O)C1CC2=C(NC3=C2ON=C3N)N1C | 7.55 |
| CC(O)C1=C(CCN)C2=C(N(C)CC2)C(=O)N1 | 7.44 |
| CC(O)C1=CC=C(NC2=NNC(O)=N2)C(=O)O1 | 7.76 |
| CC(O)C1=C(F)N2N=NC(NCCO)=C2C=C1 | 7.24 |
| CCOC1=C(F)SC2=C(NC)C(O)=NC2=C1C | 7.42 |
| CC(=O)C1=C(N)C2=C(O1)C(=C(CO)N2)[N+]([O-])=O | 7.06 |
| CC(O)C1=C(NC(=N1)C1=C(N)C=CO1)[N+]([O-])=O | 7.26 |
| CC(=O)C1=C(N(C=N)C=O)C2=C(N1)C(O)=NS2 | 7.61 |
| CC(O)C1C(O)C2=C(N1C)C(Cl)=C(N)C(=O)N2 | 7.96 |
| CC1=C2C(OC3=C2ON=C3)C2=C1C(O)=NN2 | 7.08 |
| CC(=O)C1=C(O)C(=N)N2C(CC3=C2N=NO3)=C1 | 7.02 |
| CC1C2C(O)C3=C(N2C2=CC=CN12)C(N)=CN3 | 7.00 |
| CC(O)C1=NC2=C(N1)N1CC2N2N=NC=C12 | 7.56 |
| CC(O)C1=NC2=C(N)NC(C(O)=O)=C2C=C1Br | 7.21 |
| CC1=C2C(=O)C3=C(N=CC(=O)N3)C2=C(CO)N1 | 7.14 |
| CC1=C2C(O)CC3=C(F)C=NC(N)=C3N2C=N1 | 7.20 |
| CC1=C2N3C=CC=C3NCCC2=C(N1)C(O)=O | 7.40 |
| CC1C2NC2CC2=C(N)NC3=C2N1C=C3O | 7.01 |
| CC(=O)N1N=NC2=C1C(=N)C=C(N2)C(O)=O | 7.01 |
| CC(=O)N1N=NC2=C1C(=O)NC(C(O)=O)=C2N | 7.42 |
| CC1C2NC3=C(NC(O)=C3O)C3=C2N1N=N3 | 7.03 |
| ClC1=C2CCCOC3CNC(=C23)C(=O)N1 | 7.01 |
| ClC1=C2OC3CNC4=C3N2C(=N4)C2=C1SC=C2 | 7.36 |
| ClC1=COC(=O)C2=C1C1CN2C2=C(O1)N=CO2 | 7.07 |
| CN1C2=C3C(CN2)OCC2=C(C)NC1=C32 | 7.11 |
| CN1C2=C3C(OCC3=C1C(O)C(N)=N)C(=O)N2 | 7.16 |
| CN1C2=C3N4C(CC4C2O)CC3=C(N)C1=N | 7.18 |
| CN1C2=C3N(C=C2O)N=C2C(=O)NC(=C32)C1=O | 7.04 |
| CN1C2=C(C=CN2)C2OCCN3C=C(O)C1=C23 | 7.08 |
| CN1C2=C(C=CN2)C(O)C2=C1C(=O)C(C)=CN2 | 7.11 |
| CN1C2C(=CC(O)COC2=N)C2=C1N=NS2 | 7.18 |
| CN1C2CC(OC3=C(N)C(=O)NC=C13)C=C2 | 7.36 |
| CN1C2=C(C(O)CN2)C2=C1C1OC2C1=NO | 7.55 |
| CN1C2=C(N3CC2N2C=CN=C32)C(O)=CC1=N | 7.03 |
| CN1C2=C(NC3=C(CO)NC(=O)C=C13)N=NO2 | 7.49 |
| CN1C2=C(NC=C2O)C2=C(NC1=O)N=C(O)S2 | 7.25 |
| CN1C2=C(NC(=N)C(C)=C2O)C(=O)OCC1=O | 7.40 |
| CN1C2=C(NC(=O)C(N)=C2O)C(O)COC1=O | 7.41 |
| CN1C2=C(OC3=C1OC(=O)C(N)=C3F)N=NO2 | 7.01 |
| CN1C2=NOC=C2OC2=C(C)NC(C(O)=O)=C12 | 7.67 |
| CN1CC2CCC(O2)C2=C1N=C(Cl)C(=N)N2 | 7.23 |
| CN1C=C2CNC3C4=C(N=C(N)N4)C(O)C1=C23 | 7.17 |
| CN1CC2=C(O)C3=NC(O)=CN3C3=C2C1CO3 | 7.03 |
| CN1CC2OC3=C(C=NO3)N3C=C(C#C)C1=C23 | 7.21 |
| CN1CC2OC3=C(NN=C3)N3C=C(C#N)C1=C23 | 7.30 |
| CN1CCC2=C1C(=NCC=O)N=C(O)C(N)=N2 | 7.06 |
| CN1C=CC2=C1N1C(C=O)C2N2N=NC(N)=C12 | 7.20 |
| CN1C=CC2=C1N1C(=NC(=O)C(F)=C1C)C2O | 7.45 |
| CN1C(CC2=CNC(C(O)=O)=C12)C(O)CC#N | 7.00 |
| CN1CCC2=COC=C(O)C(N)=NC(CO)=C12 | 7.22 |
| CC1=C2NC(CO)C3CN4C(CC1=C4N23)C=O | 7.70 |
| CC1=C2N=C(N)C(NCCO)=C2SC(F)=C1O | 7.39 |
| CC1C2OC3=C(C=CC(=O)N3)N1C1=C2ON=N1 | 7.75 |
| CC1C2OC3=C(NC=CC3=O)N1C1=C2NC=C1 | 7.36 |
| CC1=C2OC(COC3CN4CCN1C4=C23)C=O | 7.02 |
| CC1C2OC(=N)OCCN1C1=C2NC(=N)C=C1 | 7.21 |
| CC1C2OC(=O)CN1C1=C2C(F)=C(O)NC1=N | 7.13 |
| CC1=C2SC3=CN=NN3C2=C(NC1=O)C(O)=O | 7.10 |
| CC1=C2SC3=COC4=C3N2C2=C1NC(CO)=C42 | 7.15 |
| CC1=C(Br)C(=N)NC2=C1N1N=NC(N)=C1C2O | 7.23 |
| CC1CC2=C3N1CC1N3C3=C2NCC3OC1=O | 7.93 |
| CC1CC2=C3N1CCC3=C(O)C1=NC=CN21 | 7.32 |
| CC1CC2=CC(=N)NC(C(=O)OC=N)=C2N1C | 7.07 |
| CC1CC2=C(CO)NC(N)=C2C2=C(O1)N=NN2 | 7.36 |
| CC1CC2=C(N1C)C(=O)N=C(O)C1=C2CCN1 | 7.24 |
| CC1CC2=C(N1)C(=O)NC(C(O)=O)=C2C(C)=O | 7.13 |
| CC1CC2=C(N1)NC1=C2CC2N=C(CO)OC12 | 7.41 |
| CC1=CC2=C(NC1=O)C(O)C1C(O)CN21 | 7.13 |
| CC1CC2=CNC3=C2C(O1)=C(NC3=N)C(O)=O | 7.02 |
| CC1CC2=C(NC(C3=NC=CO3)=C2N1)N(C)C | 7.07 |
| BrC1=CN=CC2=C1OC1=NC3=C(NN=N3)OC21 | 7.17 |
| CC1CC2C(O1)C1=C3N2CC2CNC(N32)=C1O | 7.23 |
| CC1CC2C(OC3=C(O)C(N)=NN23)OCCO1 | 7.04 |
| CC1=CC2=C(S1)C1=C(NC(=N)C(=N1)C#N)C2O | 7.23 |
| CC1=CC2OC3=C(N)N=C4N3C2=C1C=C4Cl | 7.21 |
| CC1=CC=C2N(CC3=C(O)C(N)=NN23)C1=O | 7.14 |
| CC1=C(C#C)C2=C(N1)OC1=C(N2)ON=C1N | 7.10 |
| CC1=C(CC#C)C2=C(S1)C1=C(SC(=O)S1)C2O | 7.09 |
|  |  |

**Table S6.** TD-DFT results of both **MK-2** (the original, unmodified dye) and **MK2-DM1** (MK-2 with a modified donor group).

|  | **MK2-MD1** | | | **MK2** | | |
| --- | --- | --- | --- | --- | --- | --- |
| **Wavelength (nm)** | 625.3932 | 614.361 | 588.4115 | 625.3932 | 614.361 | 588.4115 |
| **Osc. Strength** | 0.0013 | 2.00E-04 | 0.0035 | 0.0013 | 2.00E-04 | 0.0035 |
| **Symmetry** | Singlet-A | Singlet-A | Singlet-A | Singlet-A | Singlet-A | Singlet-A |
| **Major contribs** | H-3->L+5 (12%),  H-3->L+8 (33%) | H-2->L+10 (20%), H-2->L+12 (10%) | H-4->L+9 (31%), H-4->L+10 (11%) | H-3->L+5 (12%), H-3->L+8 (33%) | H-2->L+10 (20%), H-2->L+12 (10%) | H-4->L+9 (31%), H-4->L+10 (11%) |
| **Minor contribs** | H-3->L+3 (2%), H-3->L+7 (2%), H-3->L+9 (2%), H-3->L+10 (3%), H-3->L+12 (4%), H-3->L+13 (2%), H-2->L+8 (2%), H-1->L+8 (4%) | H-4->L+10 (3%), H-2->L+6 (4%), H-2->L+7 (6%), H-2->L+8 (6%), H-2->L+9 (7%), H-2->L+11 (8%), H-2->L+55 (3%) | H-4->L+7 (6%), H-4->L+12 (3%), H-2->L+9 (6%), H-1->L+9 (5%), HOMO->L+9 (4%) | H-3->L+3 (2%), H-3->L+7 (2%), H-3->L+9 (2%), H-3->L+10 (3%), H-3->L+12 (4%), H-3->L+13 (2%), H-2->L+8 (2%), H-1->L+8 (4%) | H-4->L+10 (3%), H-2->L+6 (4%), H-2->L+7 (6%), H-2->L+8 (6%), H-2->L+9 (7%), H-2->L+11 (8%), H-2->L+55 (3%) | H-4->L+7 (6%), H-4->L+12 (3%), H-2->L+9 (6%), H-1->L+9 (5%), HOMO->L+9 (4%) |
